# Simple 3D printed stainless steel microreactors for online mass spectrometric analysis

**DOI:** 10.1016/j.heliyon.2019.e02002

**Published:** 2019-07-02

**Authors:** Gianmario Scotti, Sofia M.E. Nilsson, Ville-Pekka Matilainen, Markus Haapala, Gustav Boije af Gennäs, Jari Yli-Kauhaluoma, Antti Salminen, Tapio Kotiaho

**Affiliations:** a Drug Research Program, Division of Pharmaceutical Chemistry and Technology, Faculty of Pharmacy, P.O. Box 56 (Viikinkaari 5 E), FI-00014, University of Helsinki, Finland; b Laser Processing Research Group, Lappeenranta University of Technology, Tuotantokatu 2, FI-53850, Lappeenranta, Finland; c Department of Chemistry, Faculty of Science, P.O. Box 55 (A.I. Virtasen aukio 1), FI-00014, University of Helsinki, Finland

**Keywords:** Organic chemistry, Analytical chemistry, Reaction monitoring, Mass spectrometry, Stainless steel, Microreactor, 3D printing

## Abstract

A simple flow chemistry microreactor with an electrospray ionization tip for real time mass spectrometric reaction monitoring is introduced. The microreactor was fabricated by a laser-based additive manufacturing technique from acid-resistant stainless steel 316L. The functionality of the microreactor was investigated by using an inverse electron demand Diels-Alder and subsequent retro Diels-Alder reaction for testing. Challenges and problems encountered are discussed and improvements proposed. Adsorption of reagents to the rough stainless steel channel walls, short length of the reaction channel, and making a proper ESI tip present challenges, but the microreactor is potentially useful as a disposable device.

## 1 Introduction

One of the advantages of microfluidic devices for the analysis of chemical reactions is the small amount of reagents required [1]. For an experimental researcher this has value as the reagents can be expensive or need to be synthesized when not commercially available. Additionally, the reagents or reaction products may be toxic or otherwise hazardous, and minimizing their amount improves the overall chemical safety. Other advantages of microreactors compared to conventional reactors include improved temperature control due to high surface to volume ratio [2, 3], and shorter mixing times. Good control of the temperature of the reactor and low volume of reactants make also extremely exothermic reactions [2, 4] safe to study. An additional advantage of microreactors is that various analytical detection techniques can be combined with online detection [5]. Examples of reaction monitoring capabilities integrated with microreactors include mass spectrometry [6], NMR spectroscopy [7], liquid chromatography [8], and UV-Vis spectroscopy [9, 10]. Flow chemistry allows for the use of a non-destructive detection method in series with mass spectrometry. The most common mass spectrometric method used with microreactors has been electrospray ionization – mass spectrometry (ESI-MS), which has been done either by attaching the microreactor to a commercial ESI ion source [11, 12, 13, 14] or by integrating an ESI tip with the reactor [6, 15, 16, 17, 18]. A noteworthy benefit of ESI is that it is a relatively “soft” technique, making it capable to well preserve the studied analytes as intact molecules when ionizing them [19, 20]. This makes ESI particularly attractive for scenarios such as reaction mechanism investigations [21], and biopolymer and synthetic polymer studies [20, 22, 23, 24, 25].

Many of the miniaturized reactors and other microfluidic devices currently in use have been fabricated with conventional microtechnologies, which require one or more lithography steps and one or more bonding steps to produce enclosed channels and reservoirs. These technologies are also restricted to a small set of chip materials: silicon, glass, and various polymers (mostly polydimethylsiloxane and SU-8) [26]. On the other hand, 3D printing is increasingly used for the fabrication of microfluidic devices [10, 27, 28, 29, 30, 31, 32, 33, 34, 35, 36] including microreactors [13, 18]. The most common materials used by 3D printers are various polymers (e.g. polylactic acid, nylon, photocurable acrylic resins, acrylonitrile butadiene styrene [37]), but 3D printing of metals and metallic alloys is also possible with techniques such as laser additive manufacturing [38, 39, 40] (LAM, some manufacturers and authors use alternative terminology such as selective laser melting or direct metal laser sintering). LAM is a layer-wise additive manufacturing technology where a metal or alloy powder is melted by a laser beam to form 3D structures [29, 33, 39, 40]. Once one layer of material has been melted the platform with the powder is moved downwards, a new layer of powder is spread, and the laser melting process is repeated. This continues until the entire 3D object is manufactured. The movement of the laser beam is controlled by software based on an electronic 3D design of the object.

Currently, there are few microfluidic devices (with hydraulic diameter of channel equal to or less than 1 mm) made by LAM from metals, such as LAM-fabricated micro fuel cells [29, 33] liquid chromatography columns [28, 30, 41], and miniaturized reactors [42, 43]. However, 3D printed flow reactors made from 316L stainless steel for high temperature and pressure work are commercially available [44, 45]. LAM can build parts from metals and alloys such as titanium and stainless steel, including 316L — an austenitic chromium-nickel stainless steel alloy, resistant to concentrated organic and inorganic acids and bases, and other corrosive substances. 316L maintains its resistance in corrosive environments also at increased temperatures [46, 47]. For instance, 316L is resistant to a 25% solution of KOH in water at boiling point, or a 20% solution of HNO_3_ in water at 103 °C [47]. As such, it is a very attractive material for chemical microreactors. Taking in consideration the benefits of microreactors with integrated ionization sources for MS – chemical reaction studies presented and the use of 316L stainless steel for such reactors, the objective of this work is to present a simple LAM-fabricated 316L stainless steel microreactor with an integrated ESI tip, and test its applicability by analysis of an inverse electron-demand Diels-Alder and subsequent retro Diels-Alder reaction. In addition, the major aim is to discuss the challenges and problems encountered during the study and propose improvements. A preliminary study of the microreactor using corona discharge ionization, but without analysing a chemical reaction [48], as well as a preliminary report of the current study [49] have been presented previously.

## 2 Materials and methods

### 2.1 Manufacturing of the microreactor

The 3D printing instrument used for the preparation of the microreactor was custom-made by EOS GmbH, Krailling, Germany, and a similar method as previously was used to manufacture the reactors [29, 33]. Briefly, the instrument uses a 200-W continuous wave laser operating at 1070 nm wavelength. The 316L stainless steel powder had a particle size in the range of 20–50 μm (median 31 μm). After the 3D printing, the unmelted stainless steel powder was forced out of the channels by blowing with compressed air. Finally, the tip of the miniaturized reactor was manually polished to obtain a desired sharpness, i.e. small radius of curvature, so that electrospray could be performed. The estimated manufacturing cost for the microreactor presented, excluding the ESI tip sharpening process, is only about €20 in a batch of 58 pieces or more (more details in section S1).

### 2.2 Reagents and reaction conditions

*trans*-Cyclooctene-amine hydrochloride (**1**, 99%) was purchased from Sigma-Aldrich (Steinheim, Germany) and 3-[4-(6-methyl-1,2,4,5-tetrazin-3-yl)phenoxy]propan-1-amine hydrochloride (**2**, >95%) was obtained from Click Chemistry Tools (Scottsdale, USA). LC-MS Chromasolv grade acetonitrile (purity 99.9%, Honeywell, Morris Plains, USA), purified water (Milli-Q Plus, Millipore, Molsheim, France), and formic acid (98–100%, Merck, Darmstadt, Germany) were used to prepare a mixture consisting of acetonitrile:water 80:20 + 0.1 vol % formic acid. The reagents were subsequently dissolved in this solvent mixture. When this solvent mixture was infused in the absence of any analyte, it was regarded as the “background solvent” used to obtain a background/baseline signal level. To prepare samples of the reagents, *trans*-cyclooctene-amine hydrochloride (35 μg/mL, 0.13 mM) and 3-[4-(6-methyl-1,2,4,5-tetrazin-3-yl)phenoxy]propan-1-amine hydrochloride (70 μg/mL, 0.25 mM, abbreviated as tetrazine later on) were dissolved into the acetonitrile:water 80:20 + 0.1 vol % formic acid solvent mixture. The flow rate of each reagent solution and background solvent was 2.0 μL/min/syringe.

### 2.3 MS measurement conditions

**Fig. 1:**
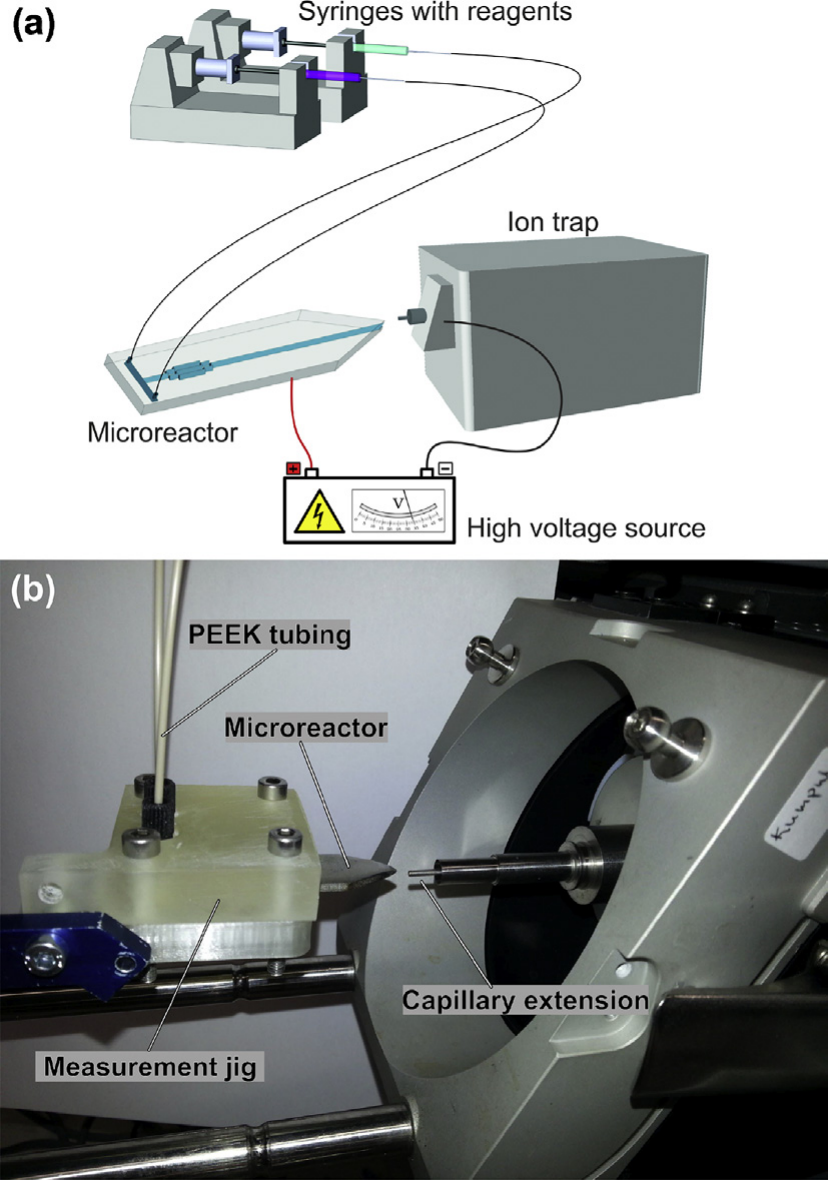
(a) A schematic presentation of the mass spectrometric measurement set-up, and (b) a photograph of the mass spectrometric measurement set-up showing the measurement jig with a stainless steel microreactor and the inlet (capillary extension) to the mass spectrometer. The jig is attached to an XYZ stage mounted on rails of an ion trap ion source frame.

The mass spectrometer used in the experiments was an Agilent 6330 ion trap mass spectrometer (Agilent Technologies, Santa Clara, USA) equipped with a capillary extension (Fig. 1). The capillary voltage of the ion trap mass spectrometer was -3 kV, the drying gas flow rate was 1 L/min and the drying gas temperature was 200 °C. The *m/z* scan range used for MS and MS^n^ measurements was 50–500. The isolation width of the precursor ion for the MS^n^ experiments was 1.0 *m/z*. In all cases except for the precursor ions *m/z* 472.3, 222.6 and 221.6, the integer value of the precursor ion was isolated, while the precursor ion was isolated with one-decimal accuracy for *m/z* 472.3, 222.6 and 221.6. MS^n^ data were recorded for 30 s per precursor ion and the obtained product ion mass spectra were averaged over this time. Due to day-to-day variance of the ion trap, the decimal of the ion *m/z* 472 and other ions can vary (e.g. see for ion *m/z* 472, Fig. 5, Fig. S4 and Fig. S12). When plotting the extracted ion profiles (EIPs), each presented ion was selected with one-decimal accuracy using a mass isolation width of ±0.5 *m/z* (1.0 *m/z* window). For processing the mass spectrometric data, the software DataAnalysis for 6300 series ion trap LC/MS version 3.4, Build 192 (Agilent Technologies, Santa Clara, USA) was used.

### 2.4 MS measurement set-up

Fig. 1 shows a schematic and an actual photograph of the mass spectrometric measurement set-up. The stainless steel microreactor was placed in a 3D printed measurement jig. An XYZ-stage (Märzhäuser Wetzlar GmbH & Co. KG, Wetzlar, Germany) attached to an ion source frame was used to position the jig with the microreactor in front of the mass spectrometer. A high voltage supply (in-house built by Dr. M. Haapala) was used to supply the microreactor with high voltage by connecting the positive outlet of the high voltage supply to the microreactor with a crocodile clamp-equipped cable, and grounding the negative outlet by connecting it to an external metal part of the mass spectrometer. The voltage of the high voltage supply was set to +5 kV (note, the capillary voltage of the MS was -3 kV) in the experiment presented (Fig. 5), which yielded a HV current of about 240 nA. The capillary current of the mass spectrometer in the presented experiment was around 130 nA. These values fall within the voltage (4.7–5.1 kV) and current ranges (HV supply current 60–270 nA and mass spectrometer capillary current 24–270 nA) typically observed with this set-up and with total flow rate of 4.0 μL/min. Reagent solutions were infused using 1-mL syringes (ID 4.6 mm, Hamilton Company Bonaduz, Bonaduz, Switzerland) in a PHD 2000 syringe pump (Harvard Apparatus, Holliston, USA). The syringes containing the reagent solutions were connected to the microreactor with PEEK capillaries (ID 250 μm, Applied Research Europe GmbH, Berlin, Germany), ferrules, nuts and sleeves (IDEX Europe GmbH, Erlangen, Germany). The flow rate of 2.0 μL/min per syringe was used for infusing the reactant solutions and background solution (i.e. the solution used to measure the background signal).

In an experiment, acquisition of mass spectra was started when infusing background solution (acetonitrile:water 80:20 + 0.1 vol % formic acid) through both syringes (Fig. S1). We noticed that it was challenging to obtain ESI; to successfully obtain ESI, the Taylor cone had to originate from a droplet formed on the tip of the microreactor's outlet. Thus, in the initial phase of each experiment it was necessary to optimize the size of this droplet, the distance between the tip of the microreactor and the capillary extension of the mass spectrometer, and the voltage of the external HV supply (see section S2 page 3). When a stable current was obtained indicating good ESI process, the syringes were exchanged to two other syringes containing the *trans*-cyclooctene and tetrazine reactant solutions, respectively, while the mass spectra acquisition was continuously on (Fig. S1). After the syringe exchange, mass spectra were recorded for at least 15 min. Between experiments the microreactor was cleaned by infusing a diluted solution of HNO_3_ in water (see Supplementary Content, section S9 for more details). Further details of the experimental procedures and conditions used in the online MS experiments are given in the section S2.

## 3 Results and discussion

**Fig. 2:**
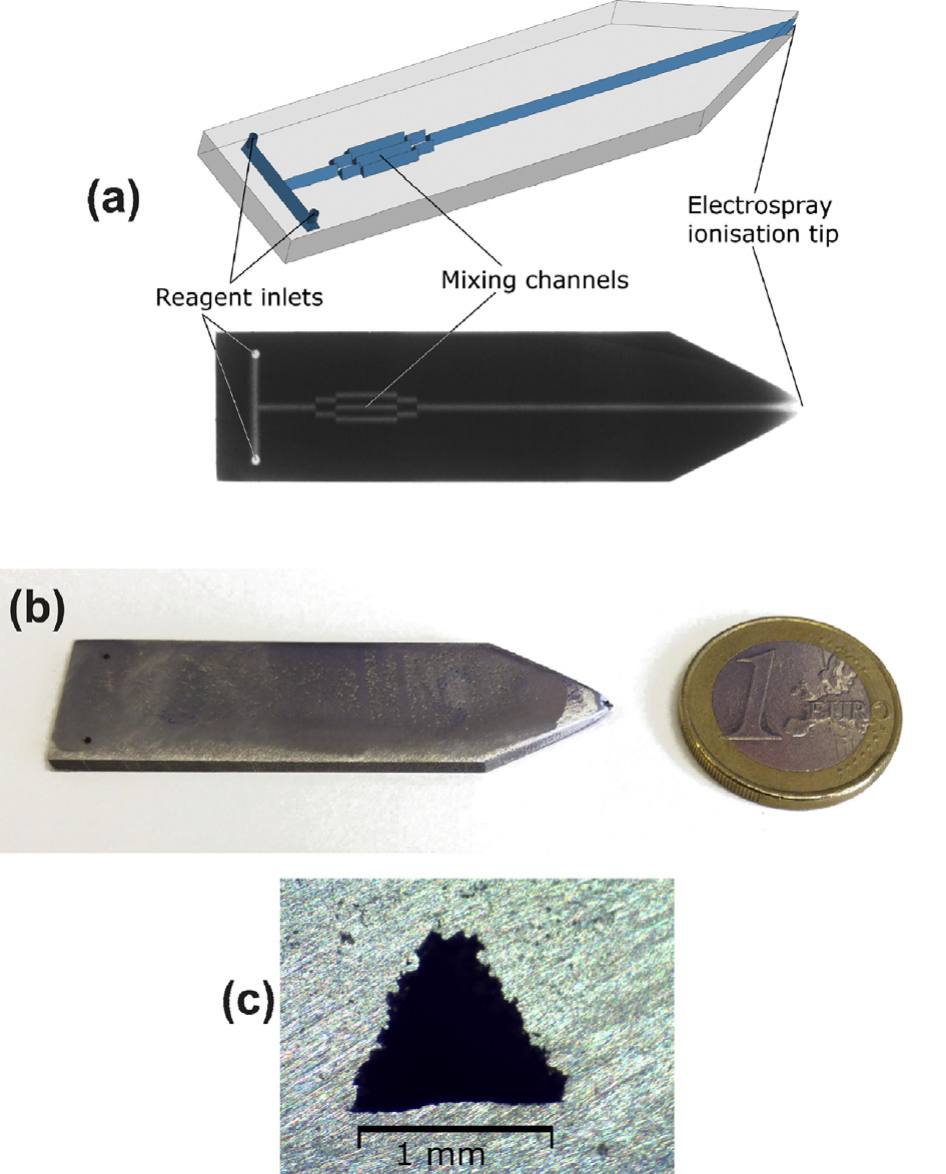
Stainless steel microreactor with an ESI tip. (a) 3D model of the microreactor and X-ray image, (b) photograph of a microreactor with a 1 euro coin for size comparison, and (c) optical micrograph of a channel cross section of a reactor with similar channel design as the microreactor in Fig. 2a-b.

Fig. 2 shows different views of a miniaturized stainless steel reactor. Fig. 2a shows a 3D model of the reactor together with an X-ray image. X-ray imaging was performed with a phoenix nanotom® (GE Digital Solutions, USA). As can be observed, the reactor channel contains overlapping prismatic channels for mixing purposes (Fig. S2), and the model and the actual final 3D printed structure agree very well. Furthermore, the high resolution X-ray images confirm that there is no occlusion of the channels. Fig. 2b shows a photograph of the microreactor next to a 1 euro coin. Fig. 2c shows an optical microscopy image of a channel cross section of a reactor with a similar channel design as the reactor in Fig. 2a-b (additional cross section images are shown in section S3, Fig. S5). As can be seen the inner walls are very rough. This roughness is related to the particle size (∼30 μm) of the stainless steel powder used in the LAM process. The root mean square (RMS) roughness of LAM-fabricated surfaces created with the same method and apparatus as the current microreactor has been measured in a previous work [33] to be R_q_ = 16.4 μm. In addition to the roughness, the inner walls are characterized by re-entrant structures — pockets in which reagents can settle producing memory effects.

The channels have been designed to have an equilateral triangular profile with a 1.2-mm side. This profile was selected due to LAM design restrictions where a hanging (unsupported) structure should not be parallel to the build platform. To achieve repeatable results, the inclination of the unsupported structure relative to the horizontal plane (i.e. the build platform) should be at least 45°. In our case the inclination of the “roof” is 60°. The triangular channel profile is the reason for the washed out look of the channels in the X-ray image.

The hydraulic diameter of channels *D*_*H*_ is an important figure of fluidic — including microfluidic — devices. It is calculated as the ratio of four times the cross sectional area *A* to the perimeter *P*, *D*_*H*_ = 4 *A/P* [50]. In our case the cross section is an equilateral triangle for which $A=\frac{\sqrt{3}}{4}a^{2}$ while the perimeter P=3a so $D_{H}$ = $\frac{a}{\sqrt{3}}$ where *a* = 1.2 mm is the side of the triangle, and the calculated hydraulic diameter *D*_*H*_ = 0.69 mm.

The volume of the microreactor was experimentally determined by the following method: the first step was to measure the weight of the empty microreactor, then water was injected into the microreactor until the channels were completely flooded, and finally the weight of the filled microreactor was measured. The difference in weight is proportional to the inner volume of the microreactor. The measured volume was ∼50 μL, in good agreement with the total inner volume of the microreactor, 51.5 μL, calculated based on the electronic 3D design.

**Fig. 3:**
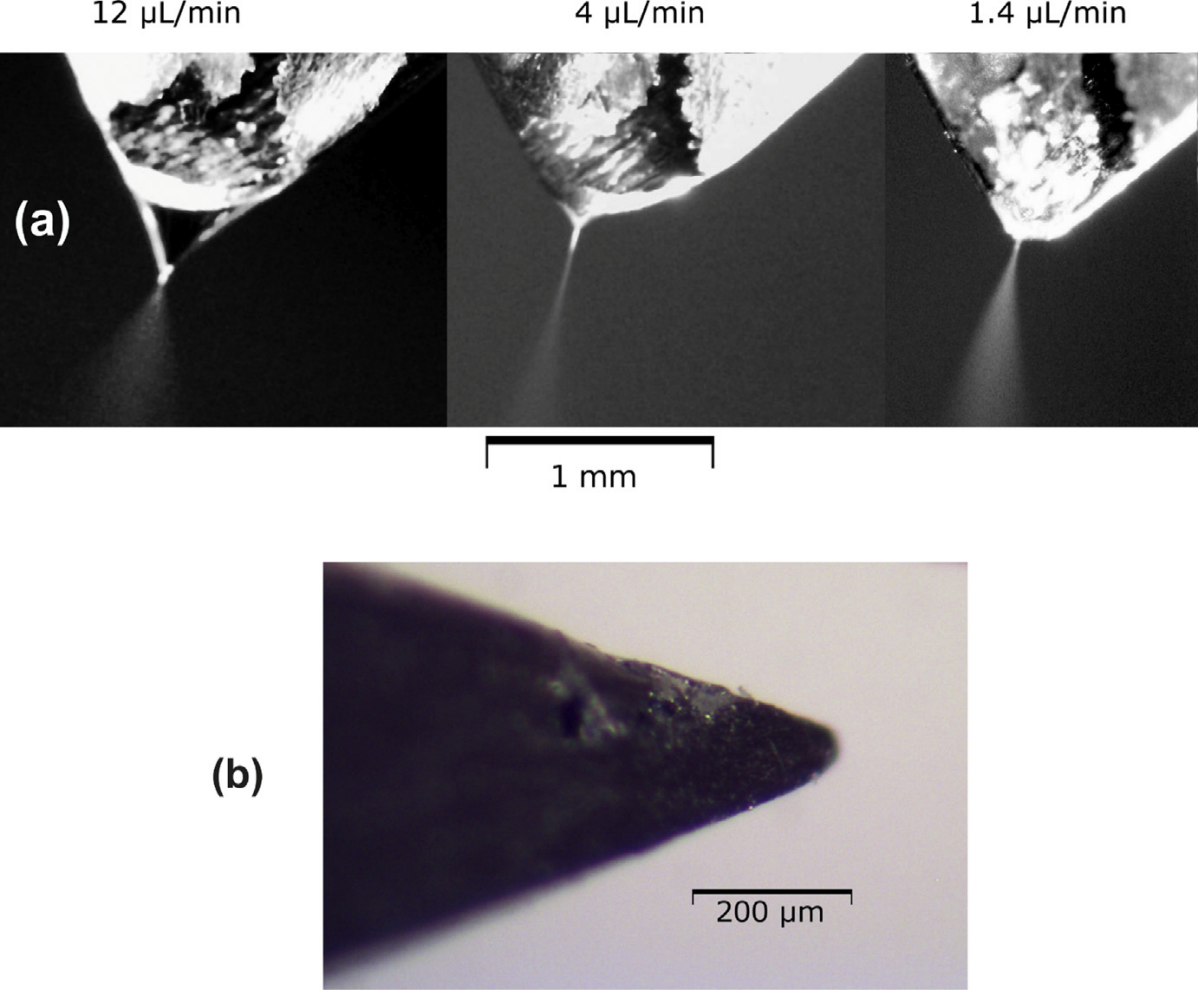
Optical microscope images of the microreactor tip. (a) Tip during electrospray with different flow rates of a solvent (an 80:20 mixture of acetonitrile:water with 0.1 vol % formic acid). (b) Side micrograph of the tip. The size measurements were obtained by placing an object of known size next to the tip (e.g. a 220-μm diameter silica capillary or an electronic calliper adjusted to 1 mm).

Before attempting to use the microreactor in conjunction with a mass spectrometer, we determined the flow rates at which electrospray can be obtained. An 80:20 mixture of acetonitrile:water with 0.1 vol % of formic acid was pumped into the microreactor, which was attached to a high voltage power supply (section S4, Fig. S6). Electrospray formation was observed starting from a total flow rate of 12 μL/min (6.0 μL/min from each reagent inlet) down to a total flow rate of 1.4 μL/min (Fig. 3). In these conditions a sufficiently small and stable Taylor cone for electrospray was obtained. However, during the experiments it was observed that formation of a droplet on the tip of the microreactor was necessary to obtain electrospray. Nevertheless, in a later mass spectrometric experiment small relative standard deviations (RSDs) of the ion currents of the extracted ion profiles of *m/z* 227 ([**1** + H]^+^) and *m/z* 246 ([**2** + H]^+^), Fig. S1b, were observed (7.4% and 3.4%, respectively), indicating a good stability of the obtained electrospray ionization. This is further discussed in section S5.

**Fig. 4:**
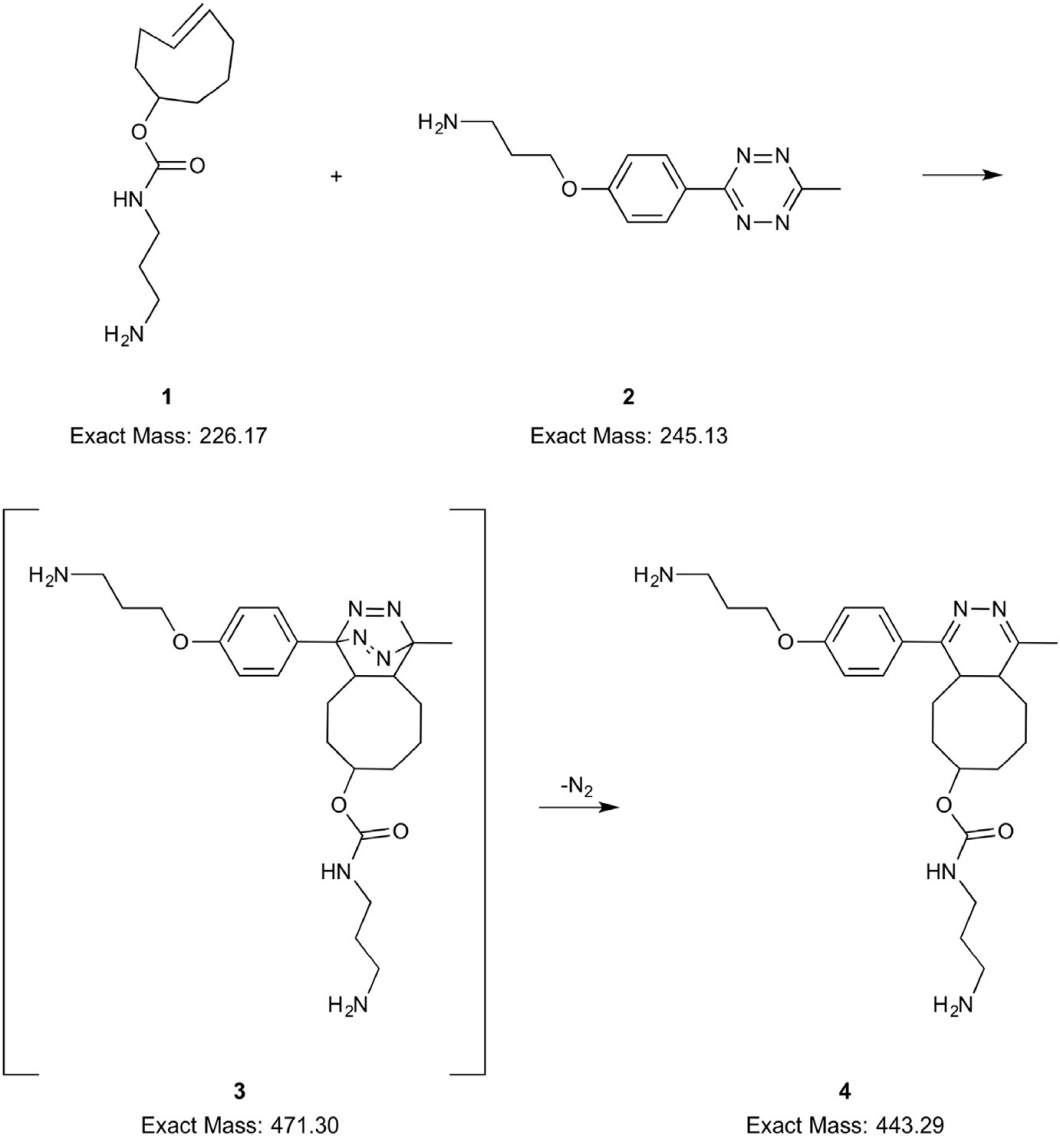
Scheme of the inverse electron-demand Diels-Alder and subsequent retro Diels-Alder reaction. The reactants are *trans*-cyclooctene, **1**, and 3-[4-(6-methyl-1,2,4,5-tetrazin-3-yl)phenoxy]propan-1-amine, **2**. Compound **3** is the reaction product of the inverse electron-demand Diels-Alder reaction before it participates as a starting material in the subsequent retro Diels-Alder reaction. Compound **4** is 4,5-dihydropyridazine, the product of the retro Diels-Alder reaction.

**Fig. 5:**
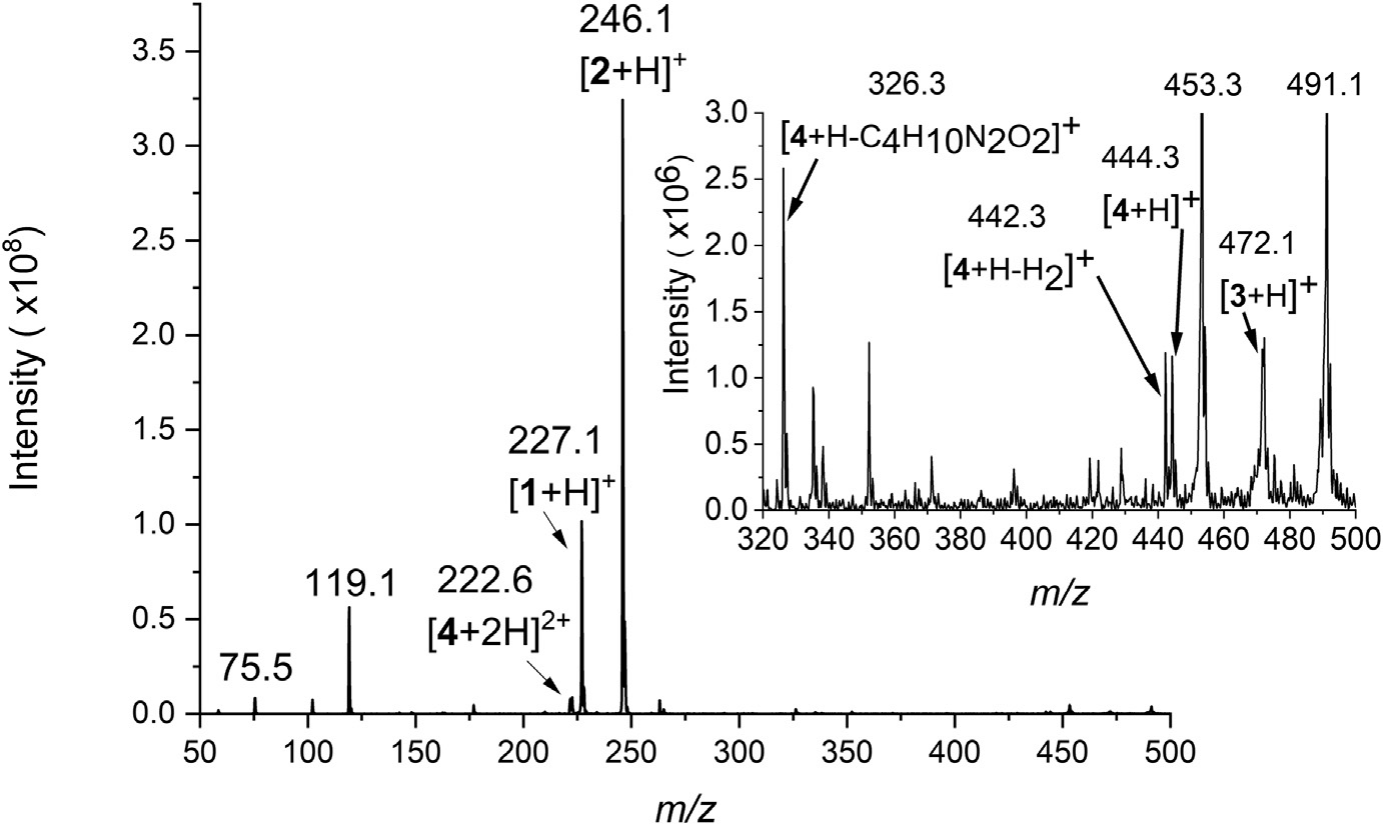
An example mass spectrum of online ESI-MS analysis of the inverse electron-demand Diels-Alder and subsequent retro Diels-Alder reaction. The reagents *trans*-cyclooctene (**1**) and tetrazine (**2**) are observed as protonated molecules at *m/z* 227, and *m/z* 246, respectively, while the reaction product 4,5-dihydropyridazine (**4**) is observed as a doubly charged ion [**4**+2H]^2+^, at *m/z* 222.6. The mass-to-charge range *m/z* 320–500 is presented in the inset with smaller y-axis scale. In the inset the reaction related ions [**4**+H–C_4_H_10_N_2_O_2_]^+^ at *m/z* 326 and [**4** + H]^+^ at *m/z* 444 are seen. In addition, ions [**4**+H–H_2_]^+^ at *m/z* 442 and [**3** + H]^+^ at *m/z* 472 are seen, which we propose also to be reaction related ions. See Sections S6 – S8 for arguments, which support these assignments.

Operation of the microreactor was tested by analyzing an inverse electron-demand Diels-Alder and retro Diels-Alder cascade of reactions (Fig. 4). An example of the results is presented in Fig. 5, which shows an ESI-MS mass spectrum of the reaction mixture. In the mass spectrum, the reagents *trans*-cyclooctene (**1**) and 3-[4-(6-methyl-1,2,4,5-tetrazin-3-yl)phenoxy]propan-1-amine (abbreviated as tetrazine from here on, **2**) are clearly observed as protonated molecules at *m/z* 227 ([**1** + H]^+^) and *m/z* 246 ([**2** + H]^+^), respectively. The doubly charged ion [**4**+2H]^2+^, *m/z* 222.6, of the product 4,5-dihydropyridazine **4** is seen with a small intensity. In the inset of Fig. 5, the protonated reaction product [**4** + H]^+^ is seen at *m/z* 444, as well as an ion at *m/z* 326, which is identified to be a fragment of the protonated reaction product [**4** + H]^+^, formed in the ionization process. Our previous study of the same reaction using a miniaturized 3D printed polypropylene reactor and online ESI-MS aided these identifications [18]. Additionally, an interesting ion for further confirmation studies is seen in the ESI mass spectrum (Fig. 5, inset), namely the ion at *m/z* 472. The ion at *m/z* 472 could be due to the cycloadduct **3** of the inverse electron-demand Diels-Alder reaction, since its *m/z* ratio matches with that of the protonated **3** ([**3** + H]^+^). A more detailed presentation on the mass spectrometry measurements and observations made e.g. discussion about the actual reaction site, and arguments for assignment of the observed ions are presented in the sections S2 and S6 – S8, respectively.

Based on the results obtained and experience gained during the experimental work, four issues were identified and are discussed below:

• Difficulties to obtain stable electrospray
• Variable delay times from start of infusion until the reagent/product signals appear
• Adsorption of reagents/products to the inner surfaces of the channels
• Reaction site uncertainties

The stability of ESI depends on the sharpness of the microreactor tip. It also depends on the size of the channel exiting at the tip: a small exit hole at the nano-ESI capillary will produce smaller droplets — enhancing ionization efficiency — compared to the larger diameter exit holes [51]. The latter issue is a problem that cannot be addressed with current LAM technology, but a better shaped channel exit might be possible to manufacture. We relied on the manually sharpened tip and the droplet at the tip deforming into a sharp Taylor cone under the effect of the applied electric field. This is a laborious process. In addition, it is well known that reactions can happen in electrospray ionization [52]. We propose that the ion detected at *m/z* 442 in the reaction mixture mass spectrum is a product formed in the electrospray ionization process (Fig. 5 inset, Fig. S7 inset on right, and discussed in section S6). One has to remember also the well-known limitations of ESI while selecting solvent systems to be used and the reactions to be studied; ESI does not generally work satisfactorily with solvents having high salt concentrations and with non-polar analytes [53] and solvents [54].

The variability of delay times from the moment of reagent infusion to the appearance of the related signals is likely due to many reasons (see section S2). One is due to the parallel channels in the mixer structure becoming filled at random times. This issue should be further investigated by designing and testing LAM microreactors with a single channel, relying on simple laminar flow mixing. In the case where the reagents are infused into a single channel, the delay between the moment reagent infusion starts and the signals appear, should only depend on the reagent solutions’ combined flow rate. Other possible reasons (e.g. partial blockage in one of the sample inlets and leakage in one of the sample introduction lines) should be possible to remove.

Another possible explanation for the delay times is the issue of compound adsorption on channel surfaces, which is directly related to the roughness of the microreactor surfaces (Fig. 2c and Fig. S5) and the number of re-entrant structures (issues discussed in further detail in section S2). With current LAM technology these properties are difficult to eliminate [27, 40]. However, roughness of LAM-fabricated channels and the size of re-entrant structures can be decreased by using a stainless steel powder with smaller particle size. Decreased particle size in conjunction with an optimized LAM process should result in smoother internal surfaces. In addition, the compound adsorption on stainless steel could be mitigated by a method similar to the one reported by Riboni et al. [55], i.e. by coating the channel walls with a thin layer of silica based material. Both approaches should be tested to decrease the memory effects.

Additional future improvements should be to increase the length of the reaction channel to provide sufficient reaction time in the channel itself, and minimize reactions in the droplet on the tip of the microreactor. This is especially important, since we cannot pinpoint the main reaction site based on the results obtained and do not know extent of the ESI contribution. The reaction channel length is easy to increase e.g. by manufacturing a microreactor with larger outer dimensions and a meandering reaction channel. Such microreactor would not be substantially different from the one presented in this study except the residence time in the channel could be increased almost arbitrarily, hence the conclusions of this work are expected mainly to apply. Minimization of the droplet size, i.e. volume for droplet reactions, could be approached by manufacturing a better defined exit hole (possible by laser drilling) at the microreactor tip.

## 4 Conclusions

In spite of the issues mentioned above, the simple stainless steel microreactors could already now be useful as disposable devices in some applications. They are not perfectly suited for studying reaction kinetics and mechanisms due to the memory effects caused by the rough surfaces in the channels, but can perform the same reaction repeatedly or continuously, especially with a much longer reaction channel and better defined tip structure for ESI. The fact that fabrication of the device requires no cleanroom work, is an additional benefit. Another benefit is the fact that the device can be easily tailored to suit specific needs through the process of rapid prototyping. Some researchers may also find very advantageous the fact that the devices are made of a mechanically robust material that can easily be heated to hundreds of degrees Celsius, to enhance the rate of reactions. For these reasons we foresee further research into improving these stainless steel microreactors.

## Declarations

### Author contribution statement

Gianmario Scotti, Sofia Nilsson: Conceived and designed the experiments; Performed the experiments; Analyzed and interpreted the data; Contributed reagents, materials, analysis tools or data; Wrote the paper.

Ville-Pekka Matilainen, Markus Haapala: Performed the experiments; Analyzed and interpreted the data; Contributed reagents, materials, analysis tools or data; Wrote the paper.

Gustav Boije af Gennäs, Jari Yli-Kauhaluoma, Antti Salminen, Tapio Kotiaho: Conceived and designed the experiments; Analyzed and interpreted the data; Wrote the paper.

### Funding statement

This work was supported by the Academy of Finland (projects 276627 and 290942).

### Competing interest statement

The authors declare no conflict of interest.

### Additional information

No additional information is available for this paper.

## Appendix A Supplementary data

18619SupportingInfoStainlessSteelMicroreactorClean 18619SupportingInfoStainlessSteelMicroreactorClean
