# Supplementary material for: Simple 3D printed stainless steel microreactors for online mass spectrometric analysis
Source: Heliyon. 2019 Jul 2;5(7):e02002. doi: 10.1016/j.heliyon.2019.e02002 (PMC6609794; doi:10.1016/j.heliyon.2019.e02002)
Supplement: 18619SupportingInfoStainlessSteelMicroreactorClean [file mmc1.pdf]

Simple 3D printed stainless steel microreactors for online mass spectrometric analysis
Supplementary Content

Gianmario Scotti[a,†*], Sofia M. E. Nilsson[a,†], Ville-Pekka Matilainen[b], Markus Haapala[a], Gustav Boije af Gennäs[a], Jari Yli-Kauhaluoma[a], Antti Salminen[b], Tapio Kotiaho[a,c*]

[a] *Drug Research Program, Division of Pharmaceutical Chemistry and Technology, Faculty of Pharmacy,*
P.O. Box 56 (Viikinkaari 5 E), FI-00014, University of Helsinki, Finland
[b] *Laser Processing Research Group, Lappeenranta University of Technology, Tuotantokatu 2, FI-53850 Lappeenranta, Finland*
[c] *Department of Chemistry, Faculty of Science, P.O. Box 55 (A.I. Virtasen aukio 1), FI-00014, University of Helsinki, Finland*

[†]*G.S. and S.M.E.N. contributed equally*
**Corresponding author*

1. Estimation of device fabrication cost 2

2. Experimental conditions/procedures with the microreactor coupled online to the ion trap mass spectrometer 3

3. Channel cross section photographs of a microreactor 12

4. Electrospray experiment set-up 13

5. Assessment of the stability of EIPs of ions connected to the Diels-Alder and subsequent retro Diels-Alder reaction 14

6. Detailed analysis of the ESI mass spectrum measured for the Diels-Alder and subsequent retro Diels-Alder reaction 15

7. MS^2 spectra 17

8. Fragmentation schemes of *m/z* 472 21

9. Cleaning of the microreactor 23

10. References 24

1. Estimation of device fabrication cost

A simple method to estimate the price per unit for the microreactor can be obtained in the following way: the build platform for the LAM system used in this study has a size of 245 mm ´ 245 mm. It is possible to place 58 microreactors horizontally on the platform, and it takes about 20 hours to fabricate them. We estimate that manufacturing of one microreactor takes around 18 g of stainless steel powder, and the price of the stainless steel powder is about €320/kg, yielding a unit-cost for the material alone of ~€6. The cost of the shielding gas is €39/hour and the depreciation of the fiber laser is about €3/hour (given that the service life is more than 50 000 hours and the price for the laser is about €150 000). Dividing the per-hour expenses by the number of microreactors, and adding the material cost of the stainless steel powder results in a per-piece price of ~€20. It should be noted that optimization of the device could slash its volume to a third without compromising functionality, hence the per-piece price could be easily brought down to about €7.

2. Experimental conditions/procedures with the microreactor coupled online to the ion trap mass spectrometer

In an experiment with the stainless steel microreactor a solvent mixture (acetonitrile:water 80:20 with 0.1 vol % formic acid) was initially infused through the microreactor representing a background measurement (Fig S1). Fig S1 shows the extracted ion profiles (EIPs) of the reagents *trans*-cyclooctene (**1**) and tetrazine (**2**), which are observed as protonated molecules at *m/z* 227 ($[\mathbf{1}+H]^+$) and at *m/z* 246 ($[\mathbf{2}+H]^+$), respectively. In addition, the EIP of the doubly charged ion ($[\mathbf{4}+2H]^{2+}$ at *m/z* 222.6) of the reaction product, 4,5-dihydropyridazine, is shown. At first the size of the droplet at the tip of the microreactor (Fig 3a) needed to be optimized (this experimental phase of the measurement is included in Fig S1a, but excluded from Fig S1b), since these were critical for successful ESI. The optimization was done by adjusting the distance of the tip of the microreactor from the capillary extension of the mass spectrometer and by adjusting the voltage of the external HV supply. If a larger droplet was observed at the tip of the microreactor, its size was minimized by blowing on it with a stream of nitrogen gas, from a separate nitrogen source. Occasionally between the reaction experiments the tip of the reactor was polished. With these methods, it was possible to fine-tune the droplet's size to obtain stable ESI with a sharp Taylor cone. At the start of the infusion of the background solvent, there was often still some *trans*-cyclooctene (**1**) and tetrazine (**2**) present in the system from a previous experiment as shown by the EIPs. This happened even when the cleaning procedure presented in the section S9 was used between the experiments. From Fig S1b it can be seen that the signals of the reagents were slowly decreasing as the background solvent infusion continued. At time point *t* = ~8 min (Fig S1b) the syringes containing the background solvent were changed to syringes containing the reagent solutions (*trans*-cyclooctene (**1**, 0.13 mM) and tetrazine (**2**, 0.25 mM), in 80:20 acetonitrile:water with 0.1 vol % formic acid). Some corona discharge and sparks were typically observed while re-optimizing the distance of the tip of the microreactor from the MS capillary extension (0.5–1 cm) to obtain optimal size of the droplet on the microreactor tip, allowing a sharp Taylor cone formation and therefore a stable electrospray ionization. When this was obtained, the typical capillary current was in the range 24–270 nA (the end plate current was typically about 70 nA). Our hypothesis is that there is some material from the previous experiments deposited on the tip, which remained there because the HNO_3 solution used for cleaning did not completely wet the tip. During the mass spectrometric experiments, the discharge can clean the microreactor tip by ablating or burning away the deposited substances. This

is seen as a clear decrease of the signals of the reagents ([**1**+H]$^+$, *m/z* 227 and [**2**+H]$^+$, *m/z* 246) in Fig S1b after the syringes were exchanged (*t* = ~8 min).

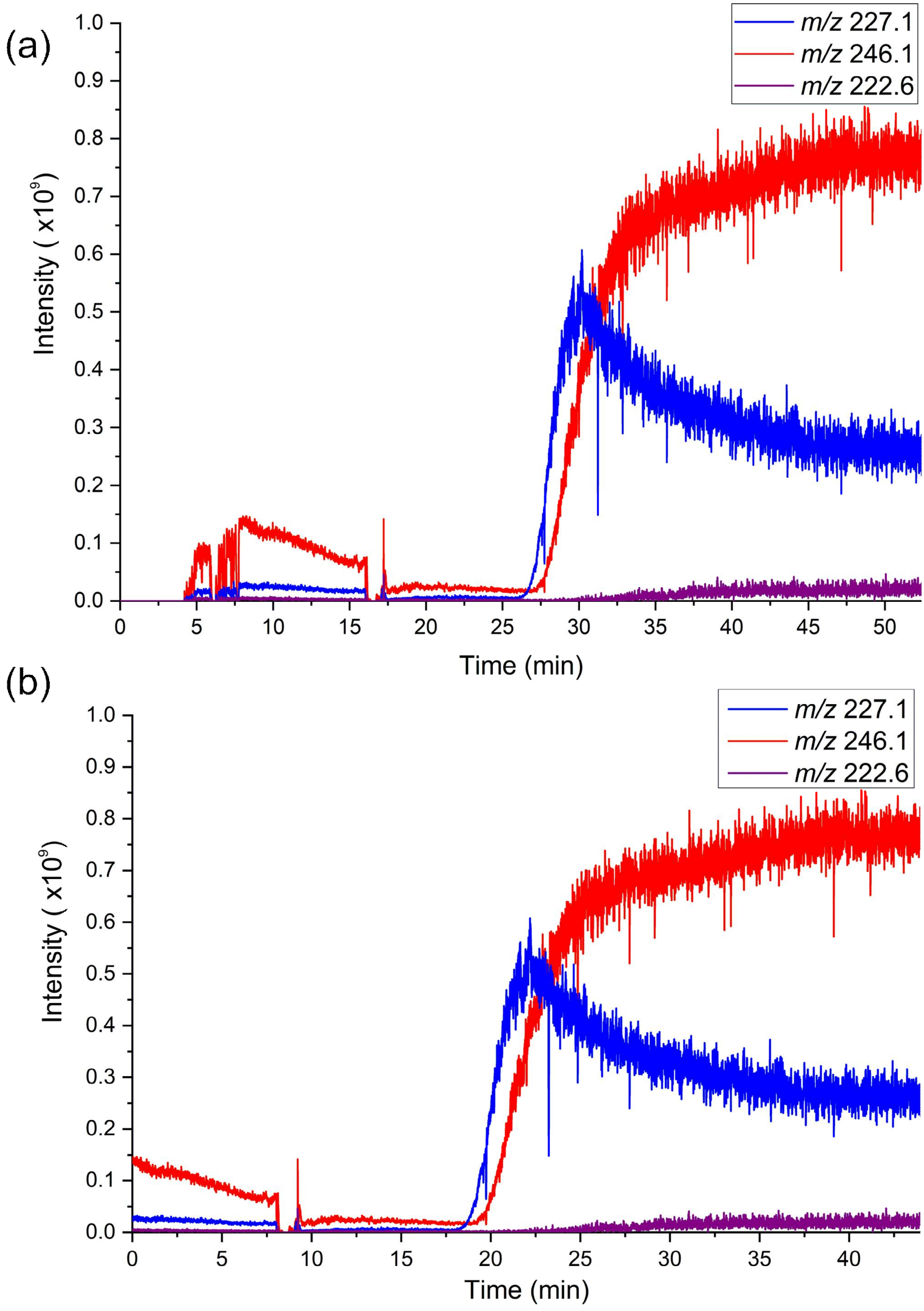
(a)
m/z 227.1
m/z 246.1
m/z 222.6
Intensity (x10⁹)
1.0
0.9
0.8
0.7
0.6
0.5
0.4
0.3
0.2
0.1
0.0
0
5
10
15
20
25
30
35
40
45
50
Time (min)
(b)
m/z 227.1
m/z 246.1
m/z 222.6
Intensity (x10⁹)
1.0
0.9
0.8
0.7
0.6
0.5
0.4
0.3
0.2
0.1
0.0
0
5
10
15
20
25
30
35
40
Time (min)

Fig S1 (on previous page). (a) The EIPs of *m/z* 227 (blue, *trans*-cyclooctene reactant $[\mathbf{1}+H]^+$), *m/z* 246 (red, tetrazine reactant $[\mathbf{2}+H]^+$), and *m/z* 222.6 (purple, doubly charged reaction product $[\mathbf{4}+2H]^{2+}$) displayed from the start of the acquisition, (b), The same EIPs as presented in Fig S1a, but with the time segment 0-8 min of Fig S1a excluded.

Ten to twelve minutes after the syringe exchange the signals of the reagents start to increase clearly (Fig S1b). This delay time is consistent with the measured volume of the microreactor (~50 µL) and a combined sample flow rate of 4.0 µL/min which give liquid flow time through the microreactor of 12.5 min. Shorter or longer, random delay times have also been observed. The shorter delay times are likely caused by the parallel branches of the mixer being filled at different times, randomly (Fig S2a). The longest delay time would be obtained in case all the branches were filled simultaneously (Fig S2b). This phenomenon has been also observed while measuring the inner volume of the microreactor; as we injected water into the microreactor, sometimes we obtained volumes as low as ~30 µL. We observed the lower measured values of internal volume mostly before starting infusion of a diluted solution of HNO_3 in water to clean the microreactor. Once the microreactor's channels have been treated with HNO_3, the measured volume was 50 µL (determined by the weighing method described in the main article), consistently with only the occasional outlier. This suggests that the problem with filling the parallel branches of the mixer results from wettability issues of metallic surfaces contaminated with organic films.[1] Once a metallic surface is treated with an oxidizing agent such as HNO_3, wetting is improved and becomes consistent. Additional delay can be caused by adsorption of the compounds to stainless steel surfaces (see below) or reasons such as partial blockage in one of the sample inlets, leakage in one of the sample introduction lines or syringe settling time – concerns of which every researcher working with miniaturized devices needs to be aware. The latter three problems could also be reasons why sometimes the signal of one of the reagents increased clearly later than that of the other one.

Formation of the product 4,5-dihydropyridazine **4** can be seen after the increase of the signal of the tetrazine **2** (*m/z* 246, Fig S1b and Fig S3a). The product formation is presented here using its doubly charged ion $[\mathbf{4}+2H]^{2+}$, since it was more intense than the singly charged ion (Fig S3b). We do not understand why the signal of *trans*-cyclooctene (**1**, *m/z* 227) starts to decrease compared to the signal of tetrazine (**2**, *m/z* 246). A stable level of the signals of the reagents and the product was only obtained long time after (~15 min) the increase of the signal of the reagents (Fig S1, S3 and S4), and it was observed that the EIPs of the protonated 4,5-dihydropyridazine $[\mathbf{4}+H]^+$ and its fragment *m/z* 326 follow well the shape of the EIP of the doubly charged ion (*m/z* 222.6) of the reaction product **4** (Fig S3b). The gradual increase of the reagent/product signals is very likely

caused by their adsorption on the rough surface of the channels. Adsorption of organic compounds on stainless steel surfaces is a known phenomenon in analytical chemistry,[2–4] and in case of the microreactor in this work the effect is exacerbated by the roughness of the channels. One cause for the long stabilization time might also be that the reaction is taking place partly in the microreactor channel and partly in the droplet on the tip of the microreactor, we cannot state what the main reaction site is (for an additional discussion about the reactivity in ESI, see Section S6).

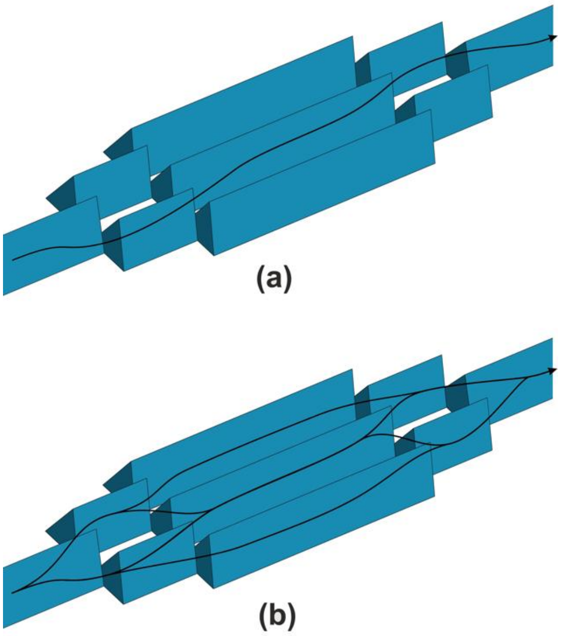

Fig S2. 3D models of the simple mixer design — overlapping prismatic channels. Illustrations of partial vs. complete filling of parallel branches of the mixer topology are also schematically presented. (a) The liquid fills only one parallel branch of the mixer. There can be other permutations of this case. (b) The mixer is completely filled.

Additionally, EIPs of ions assigned as a possible background solvent ion *m/z* 145[5] and another background ion *m/z* 203 are presented (Fig S3c, Fig S4g and Fig S4h) to show that the shapes of the EIPs of these background ions are different (after about 20-25 min) from the ions originating from the actual reaction. However, the low abundance peaks which are identified to be reaction product ions can contain also contribution from background ions, even though their EIPs (Figs S3 – S4) are similar and logical based on the expected product formation. This was seen from the measured MS^n mass spectra, which sometimes clearly contained mass peaks which could not be assigned to be formed from the expected precursor ions. The data presented in this section is from the same experiment as presented in Fig 5 and Fig S7. Structures of reaction species discussed are shown in Fig 4.

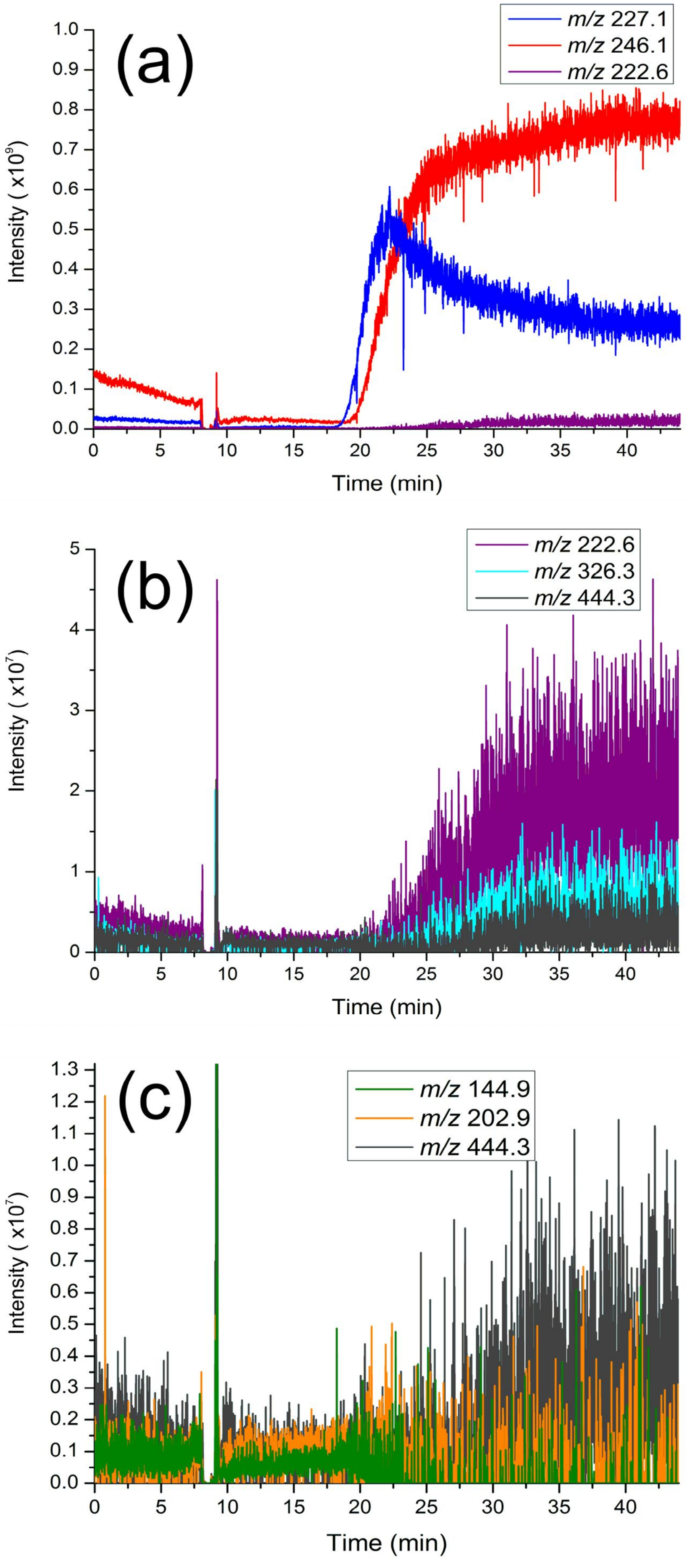
(a)
m/z 227.1
m/z 246.1
m/z 222.6
Intensity (x10^9)
Time (min)
(b)
m/z 222.6
m/z 326.3
m/z 444.3
Intensity (x10^7)
Time (min)
(c)
m/z 144.9
m/z 202.9
m/z 444.3
Intensity (x10^7)
Time (min)

Fig S3 (on previous page). (a) The EIPs of *m/z* 222.6, 227 and 246, (b) The EIPs of *m/z* 222.6, 326 and 444 and (c) The EIPs of *m/z* 145, 203 and 444. Note that the x-axis time scale is the same as in Fig. S1b. Ions presented are:

m/z 222.6 (purple, doubly charged reaction product [**4**+2H]$^{2+}$)
m/z 227 (blue, *trans*-cyclooctene reactant [**1**+H]$^{+}$)
m/z 246 (red, tetrazine reactant [**2**+H]$^{+}$)
m/z 326 (turquoise, fragment of the reaction product, [**4**+H-$C_4H_{10}N_2O_2$]$^{+}$)
m/z 444 (grey, reaction product [**4**+H]$^{+}$)

m/z 145 (green, assigned to be a background ion from solvent mixture, [5])
m/z 203 (orange, background ion)

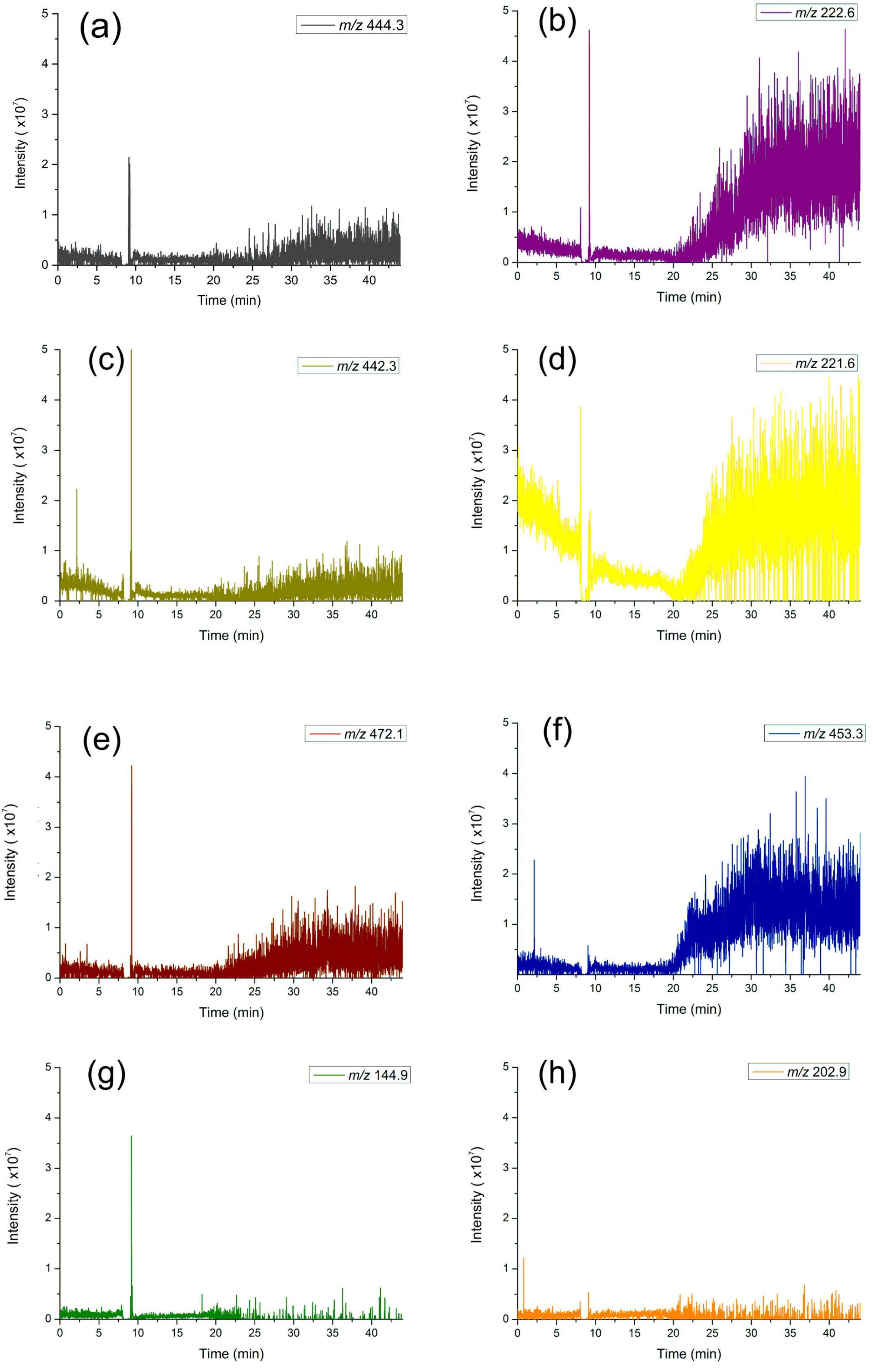
(a)
m/z 444.3
(b)
m/z 222.6
(c)
m/z 442.3
(d)
m/z 221.6
(e)
m/z 472.1
(f)
m/z 453.3
(g)
m/z 144.9
(h)
m/z 202.9
Intensity (x10^7)
Time (min)

Fig S4 (on previous page). (a) The EIP of *m/z* 444, (b) The EIP of *m/z* 222.6, (c) The EIP of *m/z* 442, (d) The EIP of *m/z* 221.6, (e) The EIP of *m/z* 472, (f) The EIP of *m/z* 453, (g) The EIP of *m/z* 145 and (h) The EIP of *m/z* 203. Note that the x-axis time scale is the same as in Fig. S1b. Ions presented are:

m/z 444 (grey, reaction product $[\mathbf{4}+H]^+$)
m/z 222.6 (purple, doubly charged reaction product $[\mathbf{4}+2H]^{2+}$)
m/z 442 (olive, assigned (see section S6) to be a reaction product related species, loss of H_2 $[\mathbf{4}+H-H_2]^+$)
m/z 221.6 (yellow, assigned (see section S6) to be doubly charged reaction product related species, loss of H_2 $[\mathbf{4}+2H-H_2]^{2+}$)

m/z 472 (dark red, assigned (see section S6) to be a reaction intermediate **3** $[\mathbf{3}+H]^+$)
m/z 453 (dark blue, assigned (see section S6) to be a reactant related ion)

m/z 145 (green, assigned to be a background ion from solvent mixture, [5])
m/z 203 (orange, background ion)

3. Channel cross section photographs of a microreactor

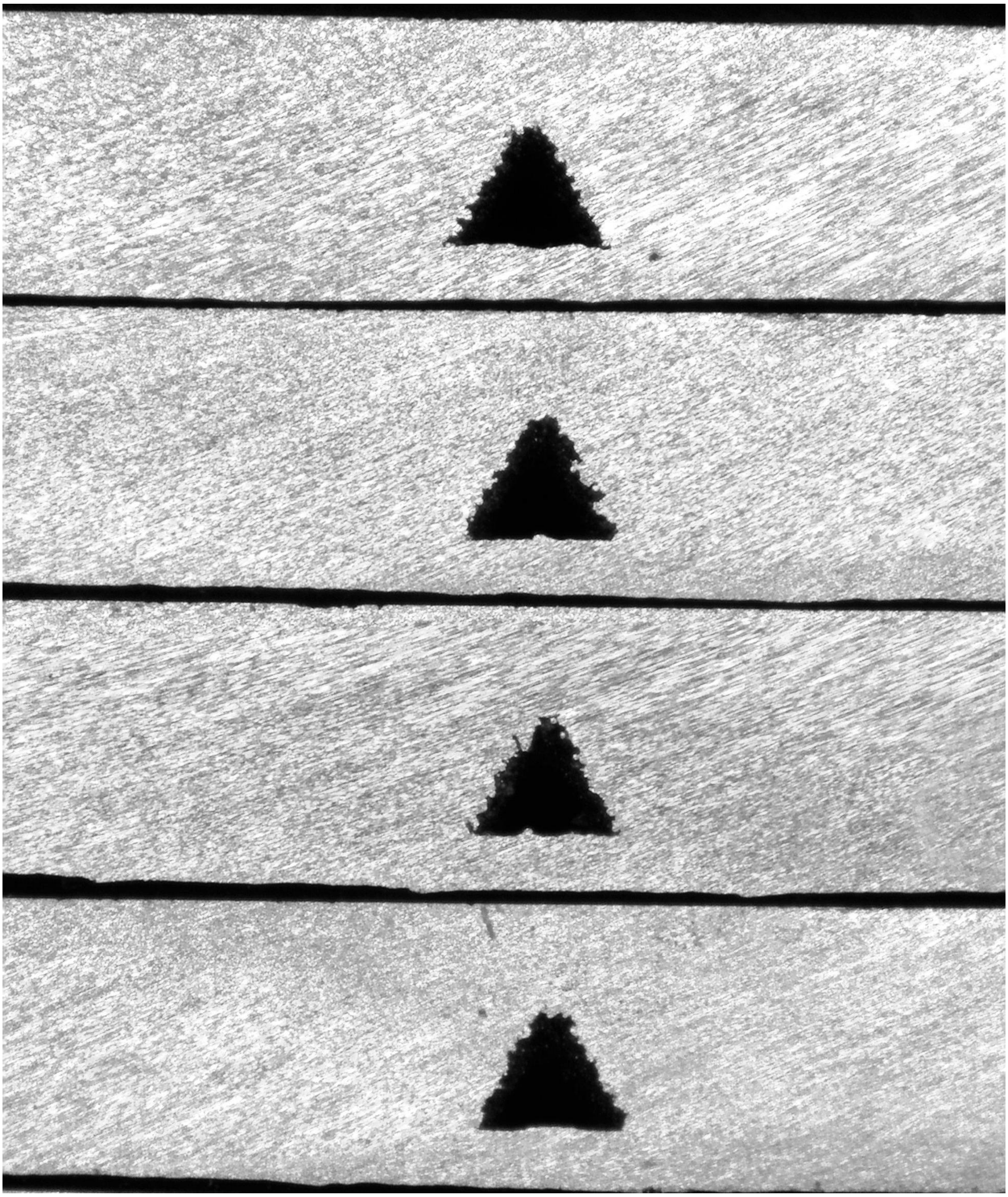

Fig S5. Channel cross section photographs of a microreactor at various points along a reactor channel path. The photographs are taken from a reactor channel having a similar design as the one used in the MS experiments. They give an idea of the roughness of the inner walls, and the geometric uniformity of the channel. The cross sections were cut at a distance of at least ~4 mm from each other. Cutting was performed by electrical discharge machining (EDM) to optimally preserve the structure of the channel.

4. Electrospray experiment set-up

Fig S6 shows the set-up used to test the electrospray capability of the microreactor. It was composed of a syringe pump infusing the 80:20 acetonitrile:water solvent with 0.1 vol % of formic acid; a PEEK T-piece to distribute the flow into both inlets of the microreactor; a high voltage power supply; a simple metallic counter-electrode, and a microscope (AM7115MZTL Dino-Lite Edge, Dino-Lite, Naarden, The Netherlands) for observation. Some additional details about the instrumentation are presented in the main article, in the MS measurement set-up section of the Materials and Methods section.

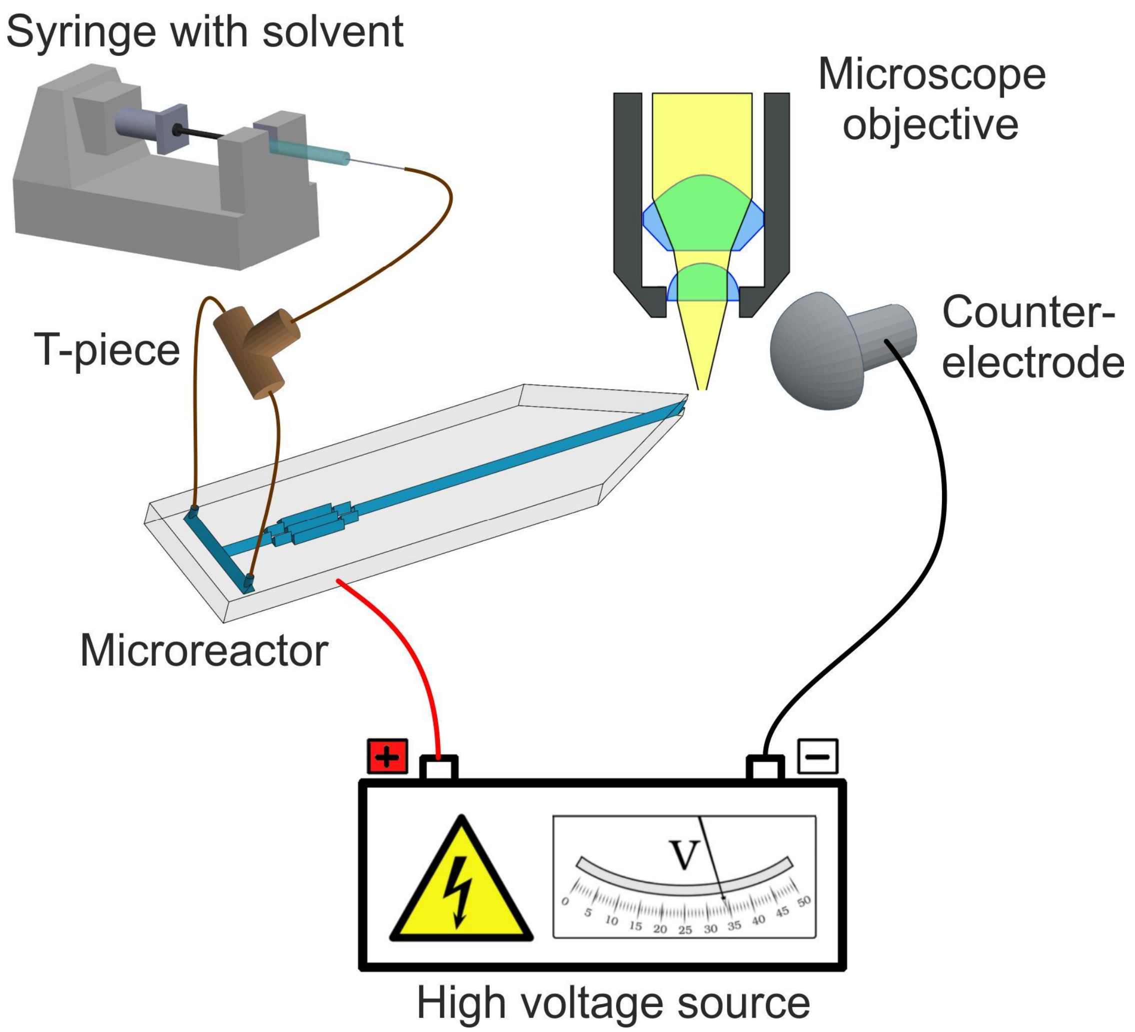

Fig S6. Schematic presentation of the electrospray experimental set-up. The distance between the microreactor tip and the counter electrode was around 3-7 mm.

5. Assessment of the stability of EIPs of ions connected to the Diels-Alder and subsequent retro Diels-Alder reaction

The stability of the ion currents of *m/z* 227 ([**1**+H]$^+$), *m/z* 246 ([**2**+H]$^+$) and *m/z* 222.6 ([**4**+2H]$^{2+}$) in Fig S1b were determined by calculating the relative standard deviations (RSD) of the extracted ion profiles for these ions from 39.0 min to the end of the acquisition (44.4 min). Small RSDs were obtained for the EIPs of *m/z* 227 and *m/z* 246 (7.4% and 3.4%, respectively), which indicate a good stability. However, the RSD for the ion *m/z* 222.6 was larger, 32%, which is explained partly by the less intensive signal. These values are well in line with the RSD values of the total ion current reported for a commercial ESI tip made of fused silica and a microchip electrospray device, which were 9.8% and 2.1%, respectively.[6]

6. Detailed analysis of the ESI mass spectrum measured for the Diels-Alder and subsequent retro Diels-Alder reaction

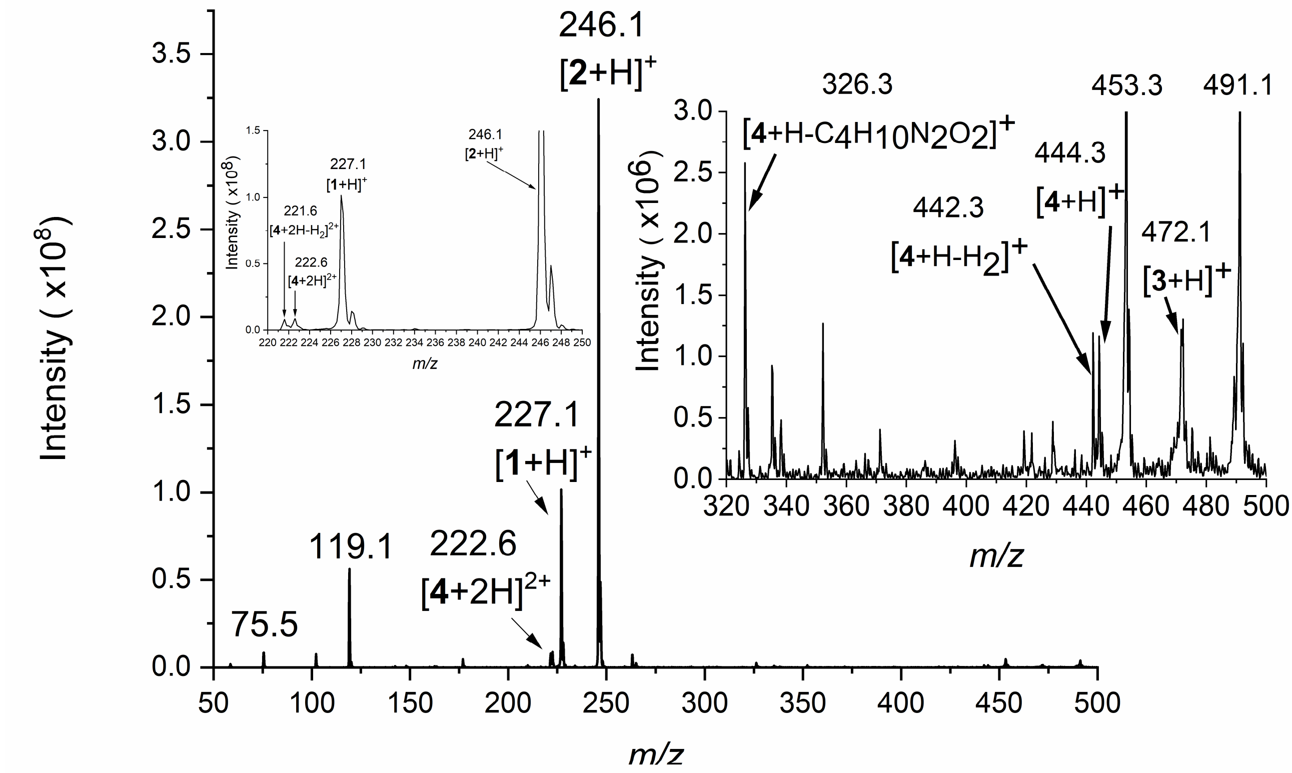

Fig S7. An example mass spectrum obtained during the online ESI-MS analysis of the inverse electron-demand Diels-Alder and subsequent retro Diels-Alder reaction, averaged over one minute at the end of the EIPs shown (43-44 min) in Figs S1b and S3 – S4. Insets with smaller y-axis scale are presented: The mass-to-charge range *m/z* 220-250 is presented in the inset (to the left), and the mass-to-charge-range *m/z* 320 – 500 is presented in the inset (to the right).

In addition to the protonated *trans*-cyclooctene (**1**, *m/z* 227), tetrazine (**2**, *m/z* 246) and the doubly charged ion, $[\mathbf{4}+2H]^{2+}$, at *m/z* 222.6 of the reaction product 4,5-dihydropyridazine (**4**) some other interesting ions are seen in the ESI mass spectrum (Fig S7). The protonated 4,5-dihydropyridazine $[\mathbf{4}+H]^{+}$ (*m/z* 444) and its fragment ion *m/z* 326 ($[\mathbf{4}+H-C_4H_{10}N_2O_2]^{+}$ [7] and Figure S11) can be seen in the mass spectrum presented in the inset of Fig S7. Two additional interesting ions for further confirmation studies can also be seen, namely ions at *m/z* 442 and *m/z* 472. The ion *m/z* 442 could be formed in the ionization process via a nominal loss of H_2 from $[\mathbf{4}+H]^{+}$ or **4**. This claim is partly supported by the fact that an ion formed by nominal loss of H_2 has been reported in literature e.g. for protonated reserpine in positive ion mode by using controlled-potential electrochemistry – electrospray emitter for ESI-MS [8]. Other formation routes are also possible, e.g. formation of [M-

H]$^+$ ion in ESI has been reported [9,10]. The fact that a doubly charged ion at *m/z* 221.6 was seen with small intensity and that the EIPs of the ion pairs *m/z* 444/222.6 and 442/221.6 (Fig S4a-d) are similar provide additional evidence that the ion *m/z* 442 and the reaction product ion *m/z* 444 are related. The ion *m/z* 472 could be due to the cycloadduct product **3** (Fig 4) of the inverse electron-demand Diels-Alder reaction, since its *m/z* ratio matches with that of the protonated **3** ([**3**+H]$^+$) and its EIP profile (Fig S4e) is similar to the EIPs of the ions *m/z* 444 and 222.6 (Fig S4a and S4b). MS^n data to support these interpretations is presented in section S7. The ions *m/z* 75 and 119 seen in the full mass spectrum (Fig 5 and Fig S7) are assigned to be fragment ions formed during the ionization process. Their formation routes and other key fragmentation routes related to the reaction are presented, along with MS^n and accurate mass data, in our previous study.[7] MS^n spectra collected during different measurement days agree well with the previously reported ones when good quality product ion mass spectra were obtained.

The additional mass peaks *m/z* 453 and 491 marked in the mass spectrum (Fig S7 inset at right) are tentatively assigned to be a reactant related ion and a background ion, respectively. The *m/z* 453 is assigned to be a reactant related ion, since its EIP (Fig S4f) follows the EIPs of the reaction product (Fig S4), and tandem mass spectrum of this ion shows a product ion *m/z* 227 (data not shown), which matches to the mass-to-charge of [**1**+H]$^+$.

7. MS^2 spectra

The identity of the reaction product ions *m/z* 221.6, 222.6, 442, 444 and 472 was studied by measuring their product ion mass spectra (Figs S8 – S12). Due to the low abundance of these ions the MS^2 mass spectra may contain contribution due to the background ions.

The precursor ion *m/z* 444 fragments (Fig S11) to one product ion, *m/z* 326, as already reported in our previous study.[7] The ion *m/z* 442 yields one product ion (Fig S10) at *m/z* 324, which is two *m/z* smaller than the product ion *m/z* 326 of the precursor ion *m/z* 444. Moreover, the precursor ion *m/z* 221.6 yields fragments (Fig S8) that either are identical (e.g. *m/z* 75, 102, and 119), one *m/z* smaller (*m/z* 162.5 vs. 163.5, a doubly charged ion) or two *m/z* smaller (*m/z* 324 vs. 326) than the fragments of the precursor ion *m/z* 222.6 (Fig S9). These facts provide additional evidence that the ions *m/z* 442 and *m/z* 444 are related.

Several ions are observed in the product ion mass spectrum of the ion *m/z* 472 (Fig S12). However, it is easily seen that a good quality product ion mass spectrum was not obtained and therefore interpretation of only the four largest mass peaks (*m/z* 241, 326, 354, and 454) was attempted. For the product ions *m/z* 326, 354 and 454 reasonable fragmentation routes (Fig S13) based on the structure assumed for the cycloadduct product **3** of the inverse electron-demand Diels-Alder reaction could be drawn. These product ions are formed by the loss of N_2+$C_4H_{10}N_2O_2$ (146), $C_4H_{10}N_2O_2$ (118), and H_2O (18) moieties, respectively. Additional evidence for the interpretation that the *m/z* 472 could be the product **3**, comes from the fact that the MS^3 mass spectrum of the product ion *m/z* 326 and the product ion *m/z* 354 of the precursor ion *m/z* 472 show a mass peak at *m/z* 269 (data not shown). Based on our earlier studies the fragment ion *m/z* 269 was formed from the precursor ion *m/z* 326 in three different MS^n experiments: 1) as a fragment of the precursor ion *m/z* 326 (MS^2 experiment), which was produced in the ionization process due to fragmentation of the protonated product 4,5-dihydropyridazine **4**, 2) as a fragment of the precursor ion *m/z* 326 (MS^3 experiment), formed by collision induced dissociation (MS^2) of the protonated product 4,5-dihydropyridazine **4**, or 3) as a fragment of *m/z* 326 (MS^3 experiment), formed by collision induced dissociation (MS^2) of the double charged ion *m/z* 222.6 ([**4**+2H]$^{2+}$).[7] For the ion *m/z* 241 we could not find reasonable formation routes based on the structure of the cycloadduct **3** and therefore it could be due to a background ion having the same *m/z* as the protonated cycloadduct **3** or alternatively it has a structure that does not correspond to that of the assumed cycloadduct **3**.

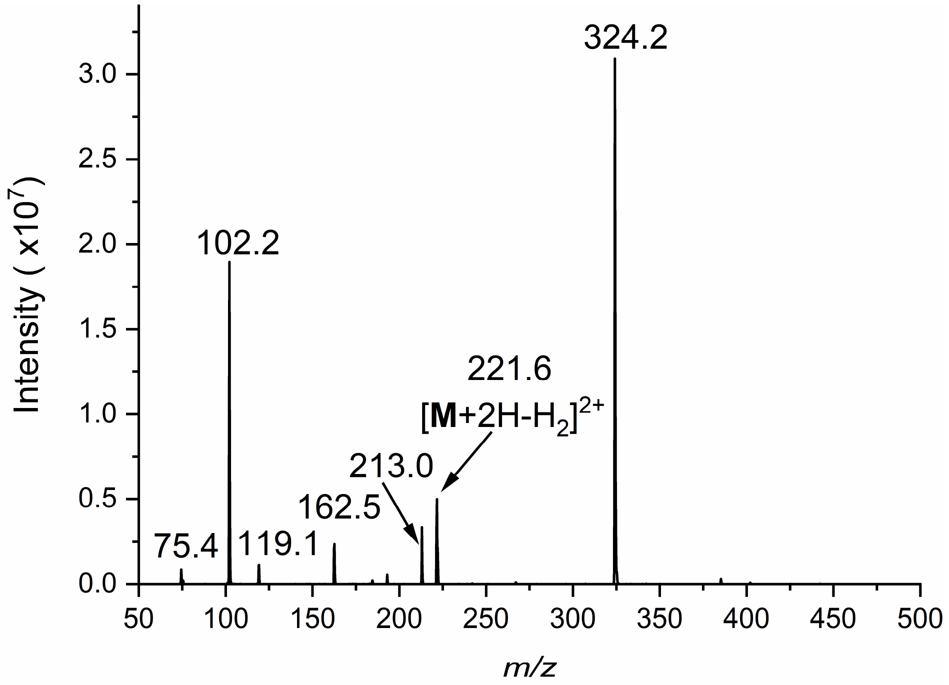

Fig S8. The MS^2 spectrum of the precursor ion *m/z* 221.6 (**M** = **4**). The fragmentation amplitude was 0.30 V. This MS^2 spectrum was recorded when the reaction was conducted online. This experiment is from another day than the experiment in Fig 5, Figs S1, S3 – S4 and S7.

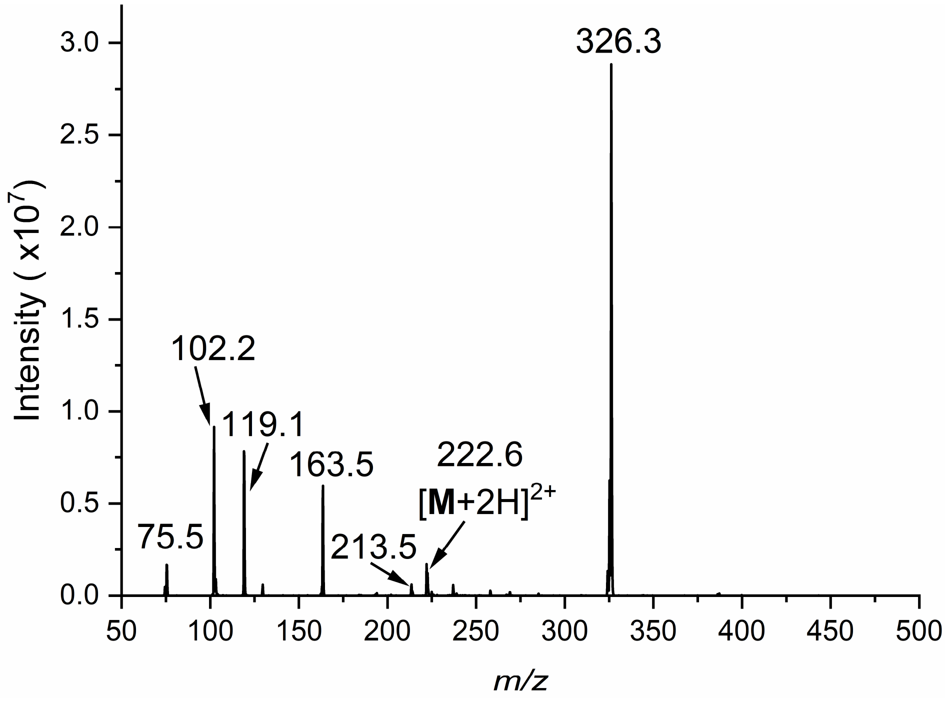

Fig S9. The MS^2 spectrum of the precursor ion *m/z* 222.6 (**M** = **4**). The fragmentation amplitude was 0.30 V. This MS^2 spectrum was recorded when the reaction was conducted online. This experiment is from another day than the experiment in Fig 5, Figs S1, S3 – S4 and S7.

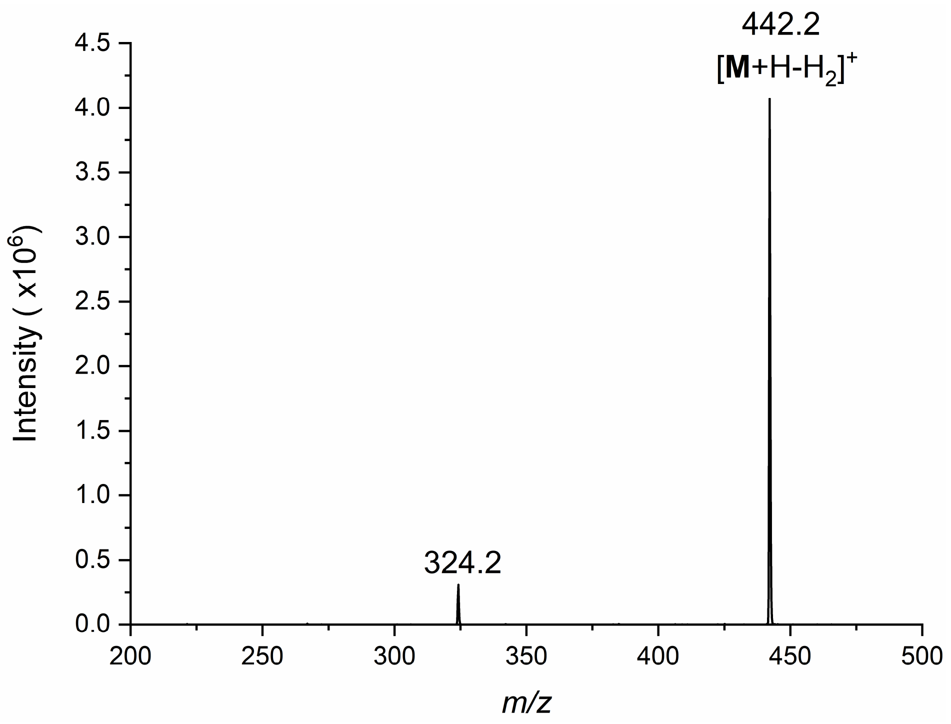

Fig S10. The MS^2 spectrum of the precursor ion *m/z* 442 (**M** = **4**). The fragmentation amplitude was 0.25 V. This MS^2 spectrum was recorded when the reaction was conducted online. This experiment is from another day than the experiment in Fig 5, Figs S1, S3 – S4 and S7.

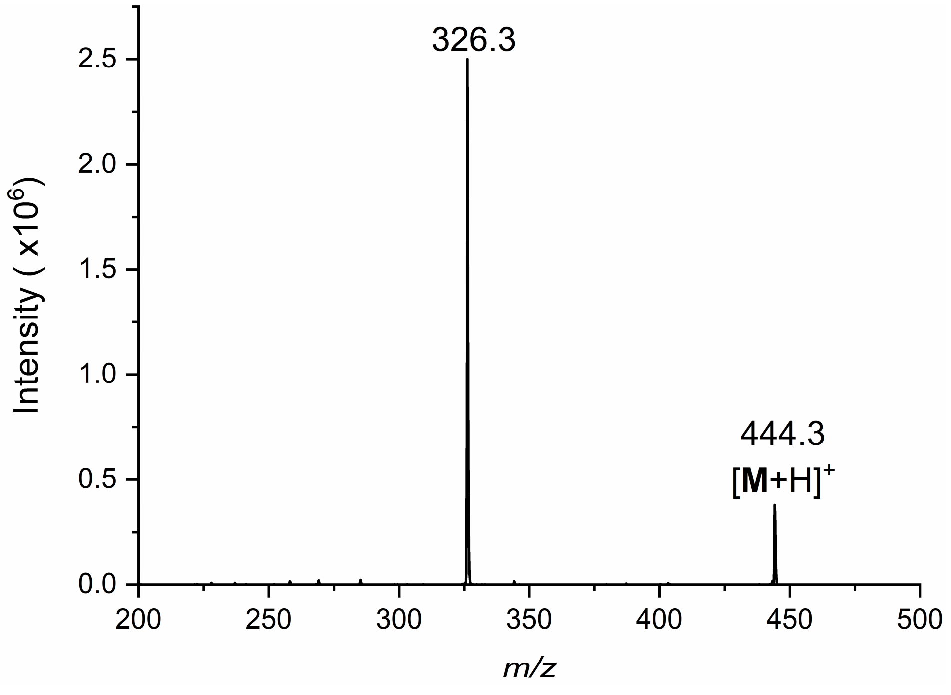

Fig S11. The MS^2 spectrum of the precursor ion *m/z* 444 (**M** = **4**). The fragmentation amplitude was 0.25 V. This MS^2 spectrum was recorded when the reaction was conducted online. This experiment is from another day than the experiment in Fig 5, Figs S1, S3 – S4 and S7.

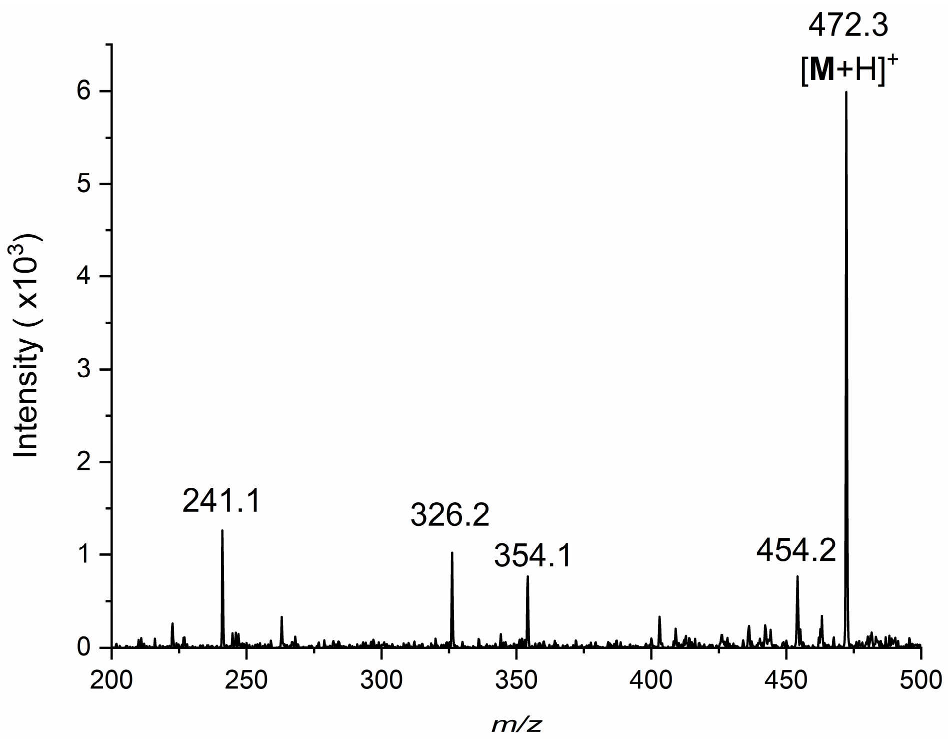

Fig S12. The MS^2 spectrum of the precursor ion *m/z* 472 (**M** = **3**). The fragmentation amplitude was 0.30 V. This MS^2 spectrum was recorded when the reaction was conducted online. This experiment is from another day than the experiment in Fig 5, Figs S1, S3 – S4 and S7.

8. Fragmentation schemes of *m/z* 472

(a)

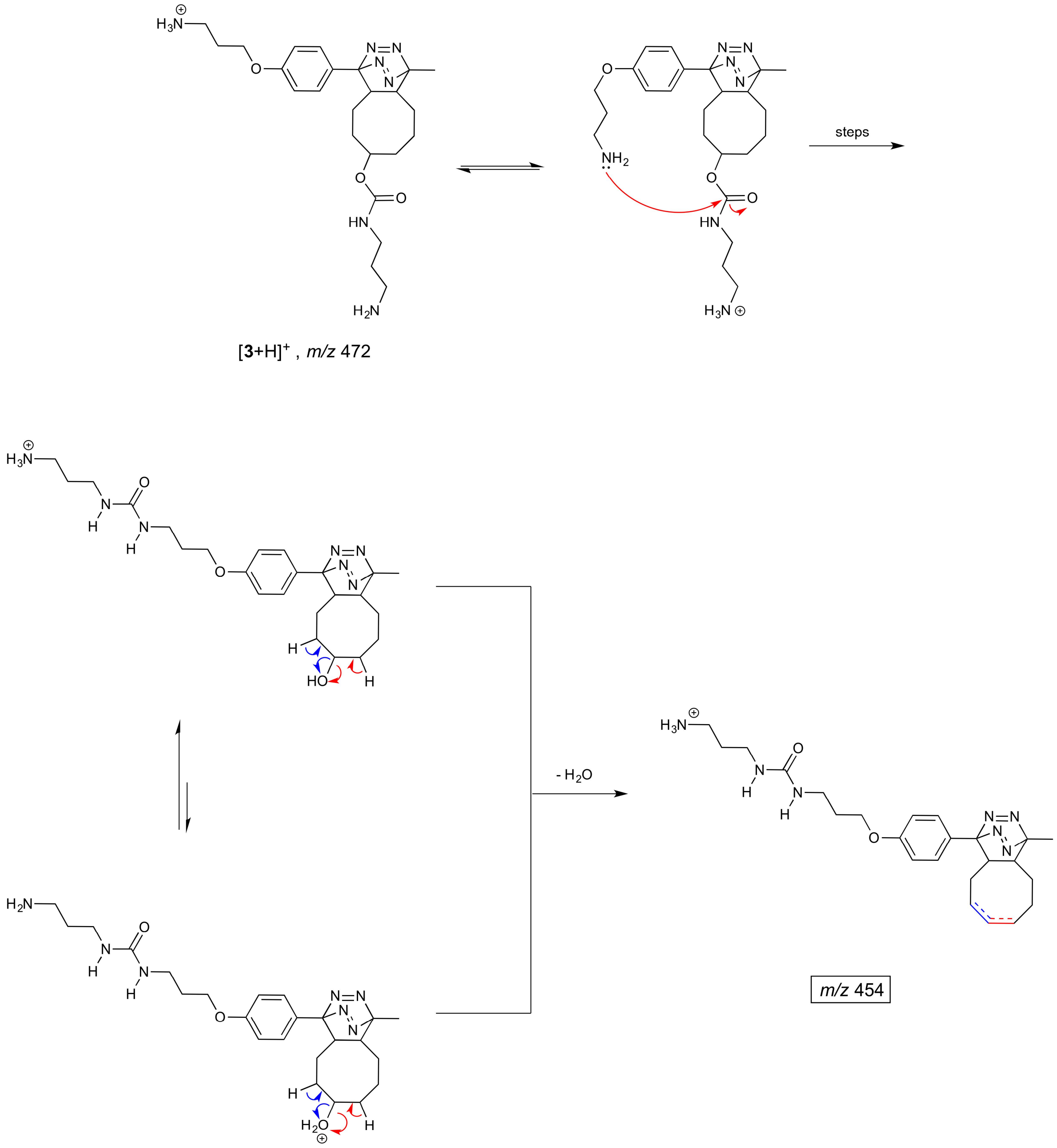

(b)

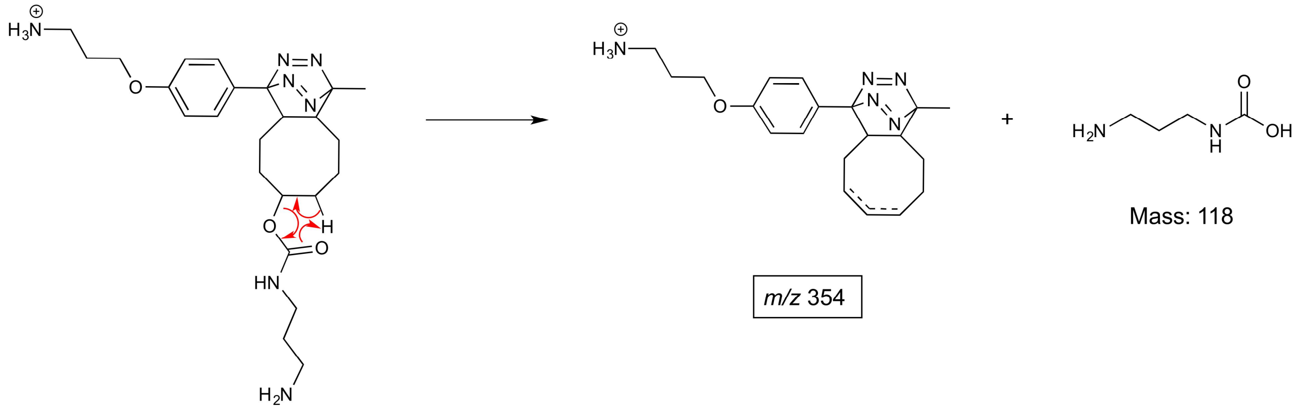

[**3**+H]$^+$, *m/z* 472

(c)

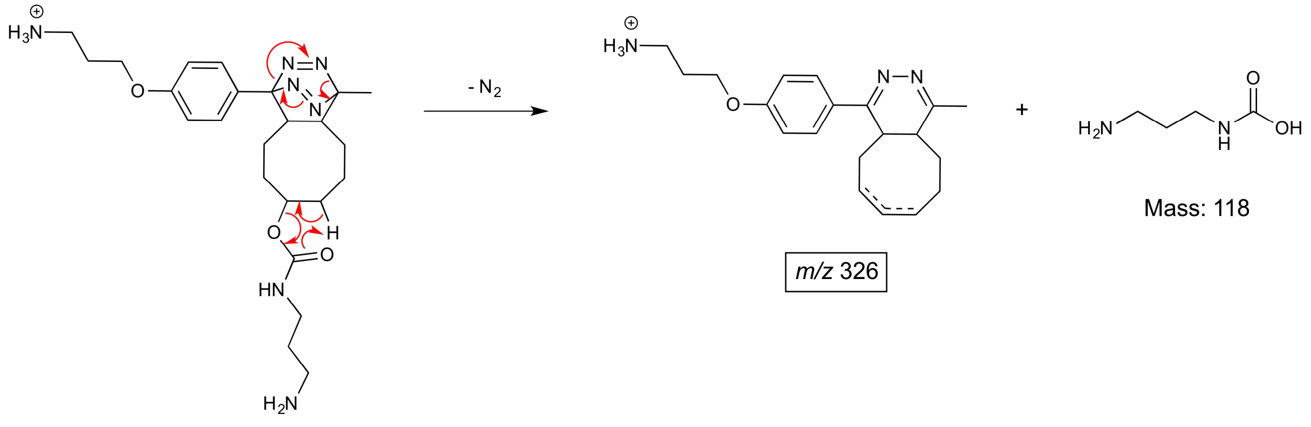

[**3**+H]$^+$, *m/z* 472

Fig S13. Fragmentation schemes for the formation of the product ions (a) *m/z* 454, (b) *m/z* 354 and (c) *m/z* 326 observed in the MS^2 mass spectrum of the precursor ion *m/z* 472, [**3**+H]$^+$ (Fig S12).

9. Cleaning of the microreactor

The cleaning procedure of the microreactor started by infusing 10 mL of a 10 wt % solution of HNO_3 in water (VWR International Oy, Helsinki, Finland) through the microreactor. After this, the microreactor was flushed with purified water (50 mL), and at the end of this flushing process, the pH of purified water exiting the microreactor was confirmed to be about 7 with the aid of pH indicator paper. The total flow rate through the microreactor in the cleaning steps was 150 µL/min. Everything exiting the microreactor during the flushing was collected in a bottle containing a 5% w/v NaOH solution in water for neutralization. The capillaries and syringes were flushed with LC-MS grade isopropanol (purity 99.9%, VWR International Oy, Helsinki, Finland), LC-MS grade acetone (purity 99.8%, Sigma-Aldrich, Steinheim, Germany), and LC-MS Chromasolv grade acetonitrile (purity 99.9%, Honeywell, Morris Plains, USA). The capillary extension of the mass spectrometer was also sonicated in these organic solvents.

10. References

[1] D.J. Trevoy, H. Johnson, The Water Wettability of Metal Surfaces, J. Phys. Chem. 62 (1958) 833–837. doi:10.1021/j150565a016.

[2] F.R. Lauritsen, A new membrane inlet for on-line monitoring of dissolved, volatile organic compounds with mass spectrometry, Int. J. Mass Spectrom. Ion Processes 95 (1990) 259–268. doi:10.1016/0168-1176(90)80025-X.

[3] N. Riboni, L. Magrini, F. Bianchi, M. Careri, A. Cappiello, Sol-gel coated ion sources for liquid chromatography-direct electron ionization mass spectrometry, Anal. Chim. Acta 978 (2017) 35–41. doi:10.1016/j.aca.2017.04.026.

[4] H.-L. Xu, Y. Li, D.-Q. Jiang, X.-P. Yan, Hydrofluoric Acid Etched Stainless Steel Wire for Solid-Phase Microextraction, Anal. Chem. 81 (2009) 4971–4977. doi:10.1021/ac900743s.

[5] B.O. Keller, J. Sui, A.B. Young, R.M. Whittal, Interferences and contaminants encountered in modern mass spectrometry, Anal. Chim. Acta 627 (2008) 71–81. doi:10.1016/j.aca.2008.04.043.

[6] G.A. Schultz, T.N. Corso, S.J. Prosser, S. Zhang, A Fully Integrated Monolithic Microchip Electrospray Device for Mass Spectrometry, Anal. Chem. 72 (2000) 4058–4063. doi:10.1021/ac000325y.

[7] G. Scotti, S.M.E. Nilsson, M. Haapala, P. Pöhö, G. Boije af Gennäs, J. Yli-Kauhaluoma, T. Kotiaho, A miniaturised 3D printed polypropylene reactor for online reaction analysis by mass spectrometry, React. Chem. Eng. 2 (2017) 299–303. doi:10.1039/C7RE00015D.

[8] V. Kertesz, G.J. Van Berkel, M.C. Granger, Study and Application of a Controlled-Potential Electrochemistry−Electrospray Emitter for Electrospray Mass Spectrometry, Anal. Chem. 77 (2005) 4366–4373. doi:10.1021/ac0503411.

[9] Y. Chai, H. Sun, J. Wan, Y. Pan, C. Sun, Hydride abstraction in positive-ion electrospray interface: oxidation of 1,4-dihydropyridines in electrospray ionization mass spectrometry, Analyst 136 (2011) 4667–4669. doi:10.1039/C1AN15129K.

[10] L. Fang, C. Dong, C. Guo, J. Xu, Q. Liu, Z. Qu, K. Jiang, Two competing ionization processes in ESI-MS analysis of *N*-(1,3-diphenylallyl)benzenamines: formation of the unusual $[M-H]^+$ ion versus the regular $[M+H]^+$ ion, Eur. J. Mass Spectrom. 24 (2018) 251–260. doi:10.1177/1469066717717228.
